# Chemotherapy and radiotherapy use in patients with lung cancer in Australia, Canada, the UK and Norway 2012–2017: an ICBP population-based study

**DOI:** 10.1136/bmjonc-2025-000800

**Published:** 2025-07-11

**Authors:** Matthew E Barclay, Sean McPhail, Shane A Johnson, Ruth Swann, Christian J Finley, John Butler, Riaz Alvi, Andriana Barisic, Damien B Bennett, Oliver Bucher, Nicola Creighton, Cheryl A Denny, Ron A Dewar, David W Donnelly, Jeff J Dowden, Laura Downie, Norah Finn, Steven Habbous, Dyfed W Huws, S Eshwar Kumar, Leon May, Carol A McClure, Bjørn Møller, David S Morrison, Grace Musto, Yngvar Nilssen, Nathalie Saint-Jacques, Sabuj Sarker, Lorraine Shack, Luc te Marvelde, Xiaoyi Tian, Robert JS Thomas, Catherine S Thomson, Richard Walton, Haiyan Wang, Tommy Hon Ting Wong, Ryan R Woods, Hui You, Bin Zhang, Georgios Lyratzopoulos, ICBP Module 9 Lung Study Group, David Baldwin

**Affiliations:** 1 Epidemiology of Cancer Healthcare & Outcomes (ECHO), Department of Behavioural Science & Health, Institute of Epidemiology & Health Care (IEHC), UCL, London, UK; 2 National Disease Registration Service, NHS England, Leeds, UK; 3 Cancer Intelligence, Cancer Research UK, London, England, UK; 4 Division of Thoracic Surgery, Department of Surgery, McMaster University, Hamilton, Ontario, Canada; 5 Canadian Partnership Against Cancer, Toronto, Ontario, Canada; 6 Department of Gynaecological Oncology, Royal Marsden Hospital NHS Trust, London, UK; 7 Epidemiology, Analytics and Surveillance, Saskatchewan Cancer Agency, Saskatoon, Saskatchewan, Canada; 8 Department of Community Health and Epidemiology, University of Saskatchewan, Saskatoon, Saskatchewan, Canada; 9 Ontario Health, Cancer Care Ontario, Toronto, Ontario, Canada; 10 Northern Ireland Cancer Registry, Queen’s University Belfast, Belfast, UK; 11 Department of Epidemiology and Cancer Registry, CancerCare Manitoba, Winnipeg, Manitoba, Canada; 12 Cancer Institute NSW, St Leonards, New South Wales, Australia; 13 Public Health Scotland, Edinburgh, UK; 14 Nova Scotia Health Cancer Care Program, Halifax, Nova Scotia, Canada; 15 Provincial Cancer Care Program, Eastern Health, St. John’s, Newfoundland and Labrador, Canada; 16 Victorian Cancer Registry, Cancer Council Victoria, Melbourne, Victoria, Australia; 17 Welsh Cancer Intelligence and Surveillance Unit, Public Health Data, Knowledge and Research Directorate, Public Health Wales, Cardiff, UK; 18 Population Data Science, Swansea University Medical School, Swansea, UK; 19 New Brunswick Cancer Network, Department of Health, Fredericton, New Brunswick, Canada; 20 Prince Edward Island Cancer Registry, Queen Elizabeth Hospital, Charlottetown, Prince Edward Island, Canada; 21 Cancer Registry of Norway, Norwegian Institute of Public Health, Oslo, Norway; 22 Cancer Advanced Analytics, Cancer Research & Analytics, Cancer Care Alberta, Alberta Health Services, Calgary, Alberta, Canada; 23 University of Melbourne, Melbourne, Victoria, Australia; 24 Cancer Control Research, BC Cancer Agency, Vancouver, British Columbia, Canada; 25 Health Analytics, Department of Health, Fredericton, New Brunswick, Canada

**Keywords:** lung cancer (non-small cell), lung cancer (small-cell), chemotherapy, radiotherapy, epidemiology

## Abstract

**ABSTRACT:**

**Background:**

International variation in lung cancer survival may be partly explained by variation in stage-specific treatment use, but relevant comparative evidence is sparse. As part of the International Cancer Benchmarking Partnership, we examined use of chemotherapy and radiotherapy in population-based cancer registry data.

**Methods:**

Linked population-based data sources were used to describe use and time to first treatment for either chemotherapy or radiotherapy in patients with lung cancer diagnosed in study periods during 2012–2017 in 16 jurisdictions of Australia, Canada, the UK and Norway.

**Results:**

There was large variation in the proportions of patients with lung cancer receiving chemotherapy (ranging from 23% in Northern Ireland to 45% in Norway) and radiotherapy (ranging from 32% in England to 48% in New South Wales and 50% in Newfoundland and Labrador). Across jurisdictions, chemotherapy use decreased steeply with increasing age, regardless of stage at diagnosis. For radiotherapy use, in stage 1–3 cancer three patterns were observed: (a) steep decrease with increasing age (UK jurisdictions, Saskatchewan-Manitoba); (b) a relatively flat pattern (Norway, Alberta, British Columbia, Atlantic Canada, New South Wales) and (c) increasing use with increasing age (Ontario).

Time to radiotherapy initiation was longer in the UK jurisdictions than elsewhere; time to chemotherapy was longer in the UK and Canadian jurisdictions except Ontario.

**Discussion:**

Use of chemotherapy and radiotherapy in patients with lung cancer varied substantially between jurisdictions during the mid-2010s within age-stage strata. Reasons for these variations are unclear. Differences in non-surgical treatment use are plausibly associated with international variation in lung cancer survival.

**WHAT IS ALREADY KNOWN ON THIS TOPIC**

- Various studies or surveillance reports have examined the management of patients with lung cancer within specific countries, but international comparisons of treatment use are limited.
- Alongside data on another seven cancer sites, two recent International Cancer Benchmarking Partnership (ICBP) studies reported on the overall use of chemotherapy and radiotherapy in patients with lung cancer as part of larger eight-site analyses.

**WHAT THIS STUDY ADDS**

- We extended previous evidence on international variation in the use of the two principal non-surgical treatments in patients with lung cancer, by additionally stratifying analyses by age-stage and sex-stage groups, and by considering treatment use jointly with time to treatment.
- We observed large interjurisdictional differences in the use of both chemotherapy and radiotherapy in patients with lung cancer during the mid-2010s.
- Such differences were apparent both between jurisdictions with relatively low versus relatively high survival (eg, England vs New South Wales), and between jurisdictions with relatively high survival (eg, stage-specific radiotherapy use in Ontario vs British Columbia).

**HOW THIS STUDY MIGHT AFFECT RESEARCH, PRACTICE OR POLICY**

- Understanding the reasons for the observed differences in management, including where possible surgery, is needed to help drive changes in practice, particularly in jurisdictions with relatively low survival.
- The reported treatment use patterns provide a critical benchmark for researchers and policymakers, both within and outside ICBP jurisdictions, to guide further research, policy actions and appropriate clinical interventions.

## Introduction

**Figure 1:**
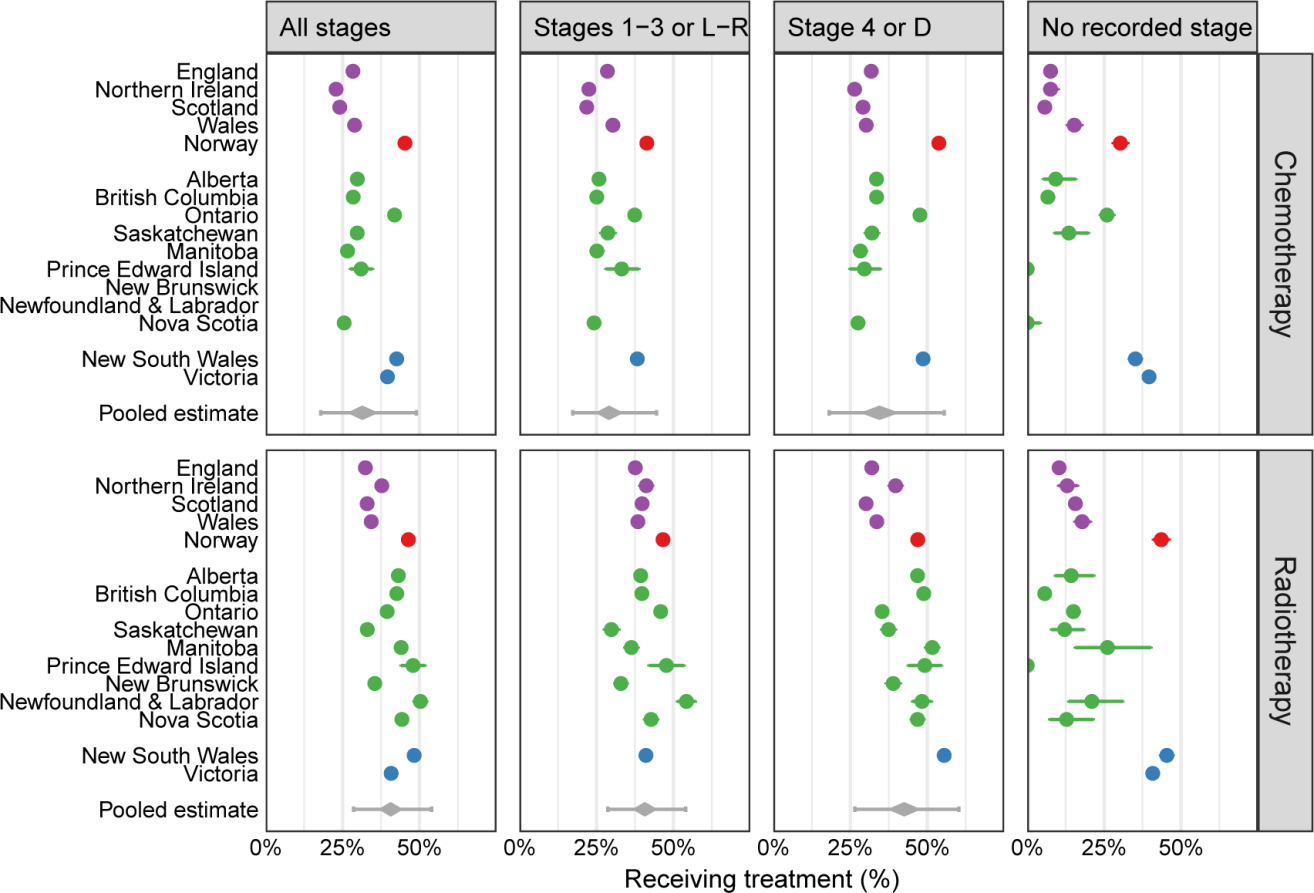
Proportion of patients with lung cancer in each jurisdiction who received chemotherapy (top) or radiotherapy (bottom) treatment, overall, for non-advanced stage, for advanced stage and for those with no recorded stage. Grey diamonds (bottom of panels) show the pooled estimate from a random-effects meta-analysis, with the widest point being the central estimate, the edges of the diamond representing 95% CIs, and the wider grey lines representing 95% prediction intervals. Same colour is used for estimates of jurisdictions of the same country. D, distant; L, localised; R, regional. See also online supplemental appendix 2 table 1.

 Lung cancer is a major public health challenge. Despite prevention efforts, millions of cases are expected to be diagnosed globally in the coming decades. Effective management can improve outcomes, but international variation in survival persists, which may partly be due to differences in treatment (online supplemental appendix 1 figure 1).^1 2^ Yet, population-based evidence on the treatment of patients with lung cancer across multiple jurisdictions is sparse.

We have recently published meta-analyses of the use of chemotherapy and radiotherapy for eight cancer sites (including lung cancer) during the mid-2010s in 16 jurisdictions of four countries.^3 4^ Here, we extend these previous analyses to examine variation in the use of chemotherapy and radiotherapy for lung cancer in specific stage strata, alongside literature-based estimates of surgery use, as a first step towards comprehensively comparing treatment patterns in this common cancer.

The study forms part of the International Cancer Benchmarking Partnership (ICBP), a collaboration of clinicians, policymakers, researchers and data experts in seven countries (Australia, Canada, Denmark, Ireland, New Zealand, Norway and the UK), which seeks to explain cancer survival differences between high-income countries with comprehensive cancer registries, similar health system expenditure and universal healthcare to help improve cancer care and outcomes globally.

## Methods

### Data

Information on chemotherapy and radiotherapy use was obtained from population-based cancer registries for patients with an incident primary diagnosis of lung cancer (International Classification of Diseases-10th revision code C34), without restriction on histological subtype. Patients were aged 15–99 years, diagnosed in study periods between 1 January 2012 and 31 December 2017, with most registries providing data for 2012–2016 inclusive (see online supplemental appendix 1 table 1). Further details of dataset specification, extraction and quality assurance have been reported previously.^3 4^

Data relate to:

- New South Wales and Victoria in Australia;
- British Columbia, Alberta, Saskatchewan, Manitoba, Ontario and Atlantic Canada (comprising Nova Scotia and Prince Edward Island in the chemotherapy analysis and, additionally, New Brunswick and Newfoundland and Labrador in the radiotherapy analysis) in Canada;
- Norway;
- England, Northern Ireland, Scotland and Wales in the UK.

Saskatchewan and Manitoba, and the Atlantic jurisdictions were considered individually in overall analysis, but due to the relatively small number of cases in each jurisdiction, they were considered jointly (as ‘SK-MB’ and ‘Atlantic Canada’, respectively) when stage groups were examined.

Two separate datasets were used, one measuring chemotherapy use and one measuring radiotherapy use. Our definition of chemotherapy aimed to include any type of systemic anticancer therapy, regardless of administration setting or administration route (see also online supplemental appendix 1 table 2 regarding possible impact of not capturing oral agents if used as monotherapy). Chemotherapy and radiotherapy treatment events were captured from routine data sources, and we assumed complete ascertainment in each jurisdiction, without censoring. This assumption was reasonable given the minimal loss to follow-up of the included registries (see online supplemental Appendix Web-Table 2.7 of the CONCORD-3 study^5^).

No direct information was available on the use of surgery, but we extracted such information from the literature for periods close to 2012–2016 for Norway,^6^ England,^7^ Scotland,^8^ Ontario,^9^ New South Wales^10^ and Victoria.^11^ We extracted information on the net survival of all patients with lung cancer, and the stage-specific net survival of patients with non-small-cell lung cancer, from previous ICBP publications.^1 2^

### Analysis

We examined use of chemotherapy and radiotherapy, defined as at least one such administration from 31 days before to 365 days after the recorded date of diagnosis in the cancer registry; and the cumulative proportion of patients receiving such treatment up to 365 days after diagnosis. The recorded diagnosis date in cancer registries is often the date of the final histological diagnosis, and the inclusion of 31 days before diagnosis is intended to capture instances where treatment started before histological confirmation; this affected fewer than 5% of registered lung cancers in any jurisdiction.

Treatment use in each jurisdiction was described by age group (15–64, 65–74, 75–84 and 85–99 years), sex (male or female) and stage. Stage-specific results relate to tumour, node, metastases (TNM) stage or, for Norway and New South Wales, summary stage. Meta-analyses of stage-specific treatment use only included jurisdictions that used TNM staging. We additionally considered stage groups, comprising ‘stages 1–3 or localised-regional (L-R)’ and ‘stage 4 or distant (D)’; these groups were considered robust based on previous analyses.^2^ Results by age group and sex were additionally stratified by stage group.

Random-effects meta-analysis characterised interjurisdictional variation in treatment use through a pooled central estimate and the 95% prediction intervals (95% PIs), which denote the range of outcomes we would expect to observe in similar jurisdictions not included in the analysis. Additionally, τ values, which represent the jurisdiction-level SD are reported as direct measures of the magnitude of interjurisdictional variability. Meta-analyses, fitted via restricted maximum likelihood using the R package metafor (V.3.4-0), assessed the log odds of treatment, which was then back-transformed to the percentage scale.^12 13^

Meta-analyses considered overall use, use by stage group (excluding Victoria) and by TNM stage (excluding Victoria, New South Wales and Norway).

For analyses by age group and by sex (with or without stratification by stage group), we considered both observed treatment use and observed between-group differences to be of key interest and did not carry out meta-analyses. Where applicable, jurisdictional patterns of treatment use were identified by visual inspection.

Finally, we used scatter plots to perform informal pairwise exploratory jurisdiction-level comparisons of use of chemotherapy, radiotherapy and, where possible, surgery, alongside comparisons of the use of these treatments with survival.

All analyses used R V.4.2.1,^14^ and the packages tidyverse and broom.^15 16^ Reporting was facilitated using the packages gt and quarto.^17 18^ Analysis code and aggregate jurisdictional data available at https://github.com/MattEBarclay/icbp_lung_2024, with aggregate jurisdictional data also given in online supplemental appendix 2.

### Patient and public involvement

Patients or the public were not involved in the design, or conduct, or reporting, or dissemination plans of this study.

## Results

### Sample description

Data were available on 279 036 patients with lung cancer for the chemotherapy analysis, and 283 773 for the radiotherapy analysis (table 1). In the chemotherapy analysis sample, almost half of all patients (49%, 135 396) were from England; 52% were male; the largest age group was 65–74 years (34%, 95 039) and 48% (132 658) were diagnosed at advanced stage, 41% (114 622) at non-advanced stage and 11% (31 756) without recorded stage. The percentage of patients without recorded stage was overall low across all jurisdictions (range 1%–14%, except Victoria which contributed no stage information, table 2A,B). The composition of the radiotherapy analysis sample was very similar to that of chemotherapy (online supplemental appendix 2 table 1).

**Table 1:** Sample description (by jurisdiction, sex, age group, stage, diagnosis year)

	Chemotherapy sample			Radiotherapy sample		
	Patients	Treated	(%)	Patients	Treated	(%)
All	279 036	88 329	(31.7%)	283 773	104 180	(36.7%)
Jurisdiction						
England	135 396	38 410	(28.4%)	135 396	43 876	(32.4%)
Northern Ireland*	5752	1316	(22.9%)	4594	1734	(37.7%)
Scotland	18 662	4496	(24.1%)	18 662	6156	(33.0%)
Wales	9365	2707	(28.9%)	9365	3214	(34.3%)
Norway	11 547	5228	(45.3%)	11 547	5356	(46.4%)
Alberta	9408	2804	(29.8%)	9408	4058	(43.1%)
British Columbia	14 739	4194	(28.5%)	14 739	6281	(42.6%)
Ontario	35 092	14 706	(41.9%)	35 092	13 857	(39.5%)
Saskatchewan	3239	964	(29.8%)	3239	1070	(33.0%)
Manitoba	3920	1043	(26.6%)	3920	1726	(44.0%)
New Brunswick	NA	NA	NA	3698	1312	(35.5%)
Newfoundland and Labrador	NA	NA	NA	2197	1105	(50.3%)
Prince Edward Island	622	193	(31.0%)	622	298	(47.9%)
Nova Scotia	4340	1107	(25.5%)	4340	1922	(44.3%)
New South Wales	16 334	6955	(42.6%)	16 334	7883	(48.3%)
Victoria	10 620	4206	(39.6%)	10 620	4332	(40.8%)
Sex						
Women	133 106	41 967	(31.5%)	135 226	47 693	(35.3%)
Men	145 930	46 362	(31.8%)	148 547	56 487	(38.0%)
Age group (years)						
15–64	69 914	35 441	(50.7%)	71 365	32 991	(46.2%)
65–74	95 039	36 106	(38.0%)	96 779	38 350	(39.6%)
75–84	82 941	15 577	(18.8%)	84 121	26 996	(32.1%)
85–99	31 142	1202	(3.9%)	31 508	5843	(18.5%)
Stage						
TNM 1	37 714	2467	(6.5%)	38 916	9736	(25.0%)
TNM 2	17 584	5607	(31.9%)	18 000	6004	(33.4%)
TNM 3	47 466	21 681	(45.7%)	48 419	25 851	(53.4%)
TNM 4	120 328	40 551	(33.7%)	122 462	42 989	(35.1%)
Localised	5188	941	(18.1%)	5188	1606	(31.0%)
Regional	6670	3761	(56.4%)	6670	3558	(53.3%)
Distant	12 330	6252	(50.7%)	12 330	6425	(52.1%)
Not recorded	31 756	7059	(22.2%)	31 788	8009	(25.2%)
Diagnosis year†						
2012	19 955	7126	(35.7%)	21 019	8639	(41.1%)
2013	58 034	18 247	(31.4%)	59 213	22 244	(37.6%)
2014	64 930	20 417	(31.4%)	66 180	24 549	(37.1%)
2015	65 172	20 651	(31.7%)	65 166	23 685	(36.3%)
2016	66 232	20 849	(31.5%)	67 482	23 648	(35.0%)
2017	4713	1039	(22.0%)	4713	1415	(30.0%)

* The difference between the chemotherapy and the radiotherapy analysis samples for Northern Ireland is due to the exclusion of 2015 radiotherapy data, owing to concerns about its completeness.

† See also online supplemental appendix 1 table 1Appendix 1 Table 1 for diagnosis year included for each jurisdiction. Only Scotland contributed data for 2017.

TNM, Tumour Node Metastases.

**Table 2:** Distribution of stage at diagnosis for lung cancer in jurisdictions using TNM (A) or summary (B) stage

(A)											
Jurisdiction	Lung cancers	TNM 1		TNM 2		TNM 3		TNM 4		Unknown	
		N	(%)	N	(%)	N	(%)	N	(%)	N	(%)
England	135 396	19 368	(14.3%)	9742	(7.2%)	26 334	(19.4%)	68 141	(50.3%)	11 811	(8.7%)
Northern Ireland (chemotherapy)*	5752	860	(15.0%)	437	(7.6%)	1320	(22.9%)	2594	(45.1%)	541	(9.4%)
Northern Ireland (radiotherapy)*	4594	675	(14.7%)	352	(7.7%)	1053	(22.9%)	2059	(44.8%)	455	(9.9%)
Scotland	18 662	2781	(14.9%)	1249	(6.7%)	3809	(20.4%)	9245	(49.5%)	1578	(8.5%)
Wales	9365	1326	(14.2%)	714	(7.6%)	2074	(22.1%)	4400	(47.0%)	851	(9.1%)
Alberta	9408	1765	(18.8%)	747	(7.9%)	1666	(17.7%)	5111	(54.3%)	119	(1.3%)
British Columbia	14 739	2298	(15.6%)	1083	(7.3%)	3005	(20.4%)	7561	(51.3%)	792	(5.4%)
Ontario	35 092	7035	(20.0%)	2780	(7.9%)	6801	(19.4%)	17 017	(48.5%)	1459	(4.2%)
Saskatchewan	3239	503	(15.5%)	111	(3.4%)	773	(23.9%)	1704	(52.6%)	148	(4.6%)
Manitoba	3920	766	(19.5%)	318	(8.1%)	792	(20.2%)	1998	(51.0%)	46	(1.2%)
Prince Edward Island	622	109	(17.5%)	37	(5.9%)	143	(23.0%)	321	(51.6%)	12	(1.9%)
New Brunswick	3698	892	(24.1%)	334	(9.0%)	776	(21.0%)	1659	(44.9%)	37	(1.0%)
Newfoundland and Labrador	2197	495	(22.5%)	167	(7.6%)	444	(20.2%)	1010	(46.0%)	81	(3.7%)
Nova Scotia	4340	903	(20.8%)	366	(8.4%)	749	(17.3%)	2236	(51.5%)	86	(2.0%)

(B)									
Jurisdiction	Lung cancers	Localised		Regional		Distant		Unknown	
		N	(%)	N	(%)	N	(%)	N	(%)
Norway	11 547	1979	(17.1%)	3259	(28.2%)	4895	(42.4%)	1414	(12.2%)
New South Wales	16 334	3209	(19.6%)	3411	(20.9%)	7435	(45.5%)	2279	(14.0%)
Victoria	10 620	0	(0.0%)	0	(0.0%)	0	(0.0%)	10 620	(100.0%)

* The difference between the chemotherapy and the radiotherapy analysis samples for Northern Ireland arises from the exclusion of 2015 radiotherapy data due to concerns about its completeness.

TNM, tumour, node, metastases.

### Chemotherapy use overall and by stage

Across all jurisdictions, the pooled estimate for chemotherapy use was 31% (95% CI 28% to 35%), with wide interjurisdictional variation (τ=0.33), ranging from 23% (Northern Ireland) to 45% (Norway) (table 3, figure 1). Chemotherapy use across all jurisdictions was similar in women and men (32% for both). There was a very steep age gradient: 51% of patients aged 15–64 years received chemotherapy, compared with 4% of those aged 85–99 years.

**Table 3:** Pooled estimates and 95% prediction intervals for the proportion of patients with lung cancer receiving chemotherapy or radiotherapy, both for all patients and for those with recorded stage 1–3 or stage 4 cancer

Treatment	Pooled estimate %	(95% CI)	95% prediction interval*	τ, log-odds scale†	I^2^‡	Observed jurisdictional range
Chemotherapy						
All stages	31.4%	(27.7% to 35.3%)	17.9% to 49.0%	0.334	99.7	22.9%–45.3%
Stages 1–3 or L-R	29.1%	(25.8% to 32.6%)	17.3% to 44.5%	0.300	99.2	21.9%–41.4%
Stage 4 or distant	34.4%	(29.8% to 39.4%)	18.1% to 55.5%	0.386	99.6	26.4%–53.8%
Stage 1	4.8%	(3.4% to 6.8%)	1.3% to 15.8%	0.567	98.0	0.0%–11.7%
Stage 2	31.3%	(26.8% to 36.1%)	17.0% to 50.2%	0.344	96.7	19.6%–46.8%
Stage 3	44.2%	(40.0% to 48.5%)	29.1% to 60.5%	0.285	98.4	34.7%–60.3%
Radiotherapy						
All stages	40.6%	(37.7% to 43.6%)	28.6% to 54.0%	0.246	99.5	32.4%–50.3%
Stages 1–3 or L-R	40.7%	(37.7% to 43.8%)	28.7% to 54.0%	0.243	98.9	29.8%–54.2%
Stage 4 or distant	42.5%	(38.5% to 46.6%)	26.5% to 60.3%	0.327	99.4	30.1%–55.5%
Stage 1	23.8%	(19.9% to 28.2%)	11.0% to 44.2%	0.414	98.6	12.3%–40.2%
Stage 2	33.6%	(31.7% to 35.6%)	27.4% to 40.5%	0.127	79.8	26.1%–43.7%
Stage 3	57.1%	(52.5% to 61.6%)	38.6% to 73.9%	0.335	98.6	41.8%–73.9%

* Prediction intervals show the range within which 95% of new jurisdictions are expected to fall; they incorporate both the uncertainty around the pooled estimate and the variability across the included jurisdictions.

† The estimated SD of the included jurisdictional estimates, directly measuring the spread across jurisdictions.

‡ The proportion of total variation not due to sampling variation.

L, localised; R, regional.

**Figure 2:**
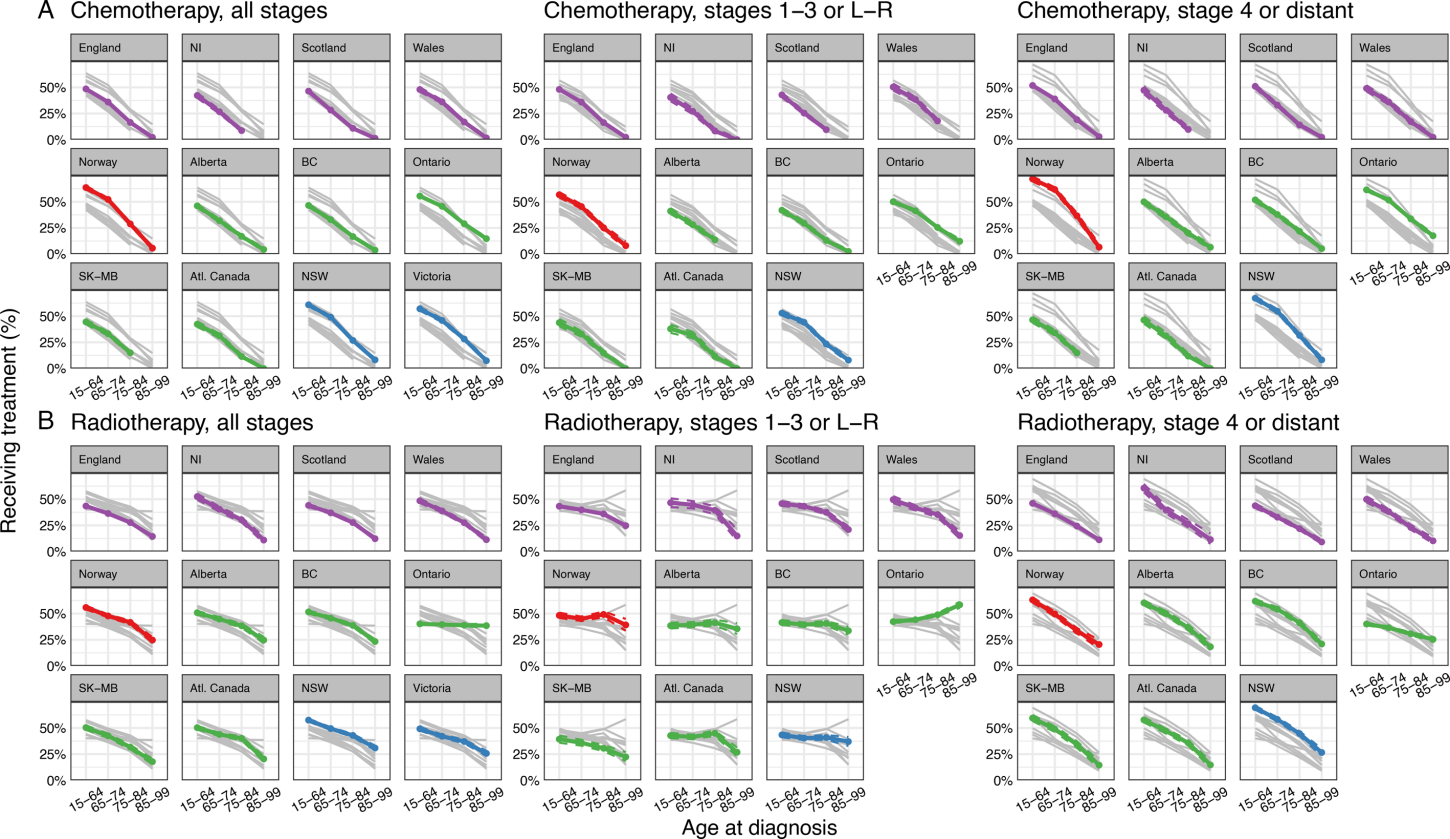
(**A–B**) Proportion of patients treated with chemotherapy (A, top) and radiotherapy (B, bottom) by age, for all stages (left), non-advanced stage (middle) and advanced stage (right). Coloured lines show jurisdictional results, with jurisdictions within the same country having the same colour lines. Dashed vertical lines show 95% CIs, noting that they often align closely to central estimates, making them hard to decipher. Grey lines show results for all other jurisdictions for ease of comparisons. Proportions based on counts of between 1 and 9 are not shown. Atl. Canada, Atlantic Canada; BC, British Columbia; L, localised; NI, Northern Ireland; NSW, New South Wales; R, regional; RT, radiotherapy; SK-MB, Saskatchewan-Manitoba. See also online supplemental appendix 2 table 2.

Considering stage-specific results, highest use was for patients with TNM 3 lung cancer, of whom 44% received chemotherapy (95% CI 40% to 49%, table 3, online supplemental appendix 1 figure 2), compared with 35% (95% CI 30% to 40%) of those with TNM 4 (or distant), 31% (95% CI 27% to 36%) of those with TNM 2 and 5% (95% CI 4 to 7%) of those with TNM 1 cancer. The magnitude of stage-specific interjurisdictional variation was similar to that observed overall, except for TNM 1 cancer where interjurisdictional variation was considerably larger (τ=0.55).

### Radiotherapy use overall and by stage

The pooled estimate for radiotherapy use was 41% (95% CI 38% to 44%), with wide interjurisdictional variation (τ=0.25), ranging from 32% (England) to 50% (Newfoundland and Labrador) (table 3, figure 1). Radiotherapy use across all jurisdictions was lower in women than men (35% vs 38%, respectively). There was a steep age gradient: 46% of patients aged 15–65 years received radiotherapy, compared with 19% in patients aged 85–99 years.

Considering stage-specific results, 57% of patients with TNM 3 cancer received radiotherapy (95% CI 53% to 62%, table 3, online supplemental appendix 1 figure 2), compared with 42% (95% CI 38% to 46%) of those with TNM 4 (or distant), 33% (95% CI 31% to 36%) of those with TNM 2 and 24% (95% CI 20% to 28%) of those with TNM 1 lung cancer. Other than for TNM 2 cancer (where interjurisdictional variation was relatively small, τ=0.14), stage-specific interjurisdictional variation in use of radiotherapy was generally higher within stage strata than observed overall. Considering the use of radiotherapy by stage category, we observe greater interjurisdictional variation for use of radiotherapy in stage 4 compared with stages 1–3 disease (τ 0.33 vs 0.24, respectively, table 3).

### Missing stage and treatment

For both chemotherapy and radiotherapy, jurisdictional patterns of use in patients with missing stage were generally concordant with those observed overall (figure 1, online supplemental appendix 1 figure 2).

### Chemotherapy use by age and stage

There were steep decreases in chemotherapy use with increasing age across the jurisdictions (figure 2A). Among jurisdictions with over 10 000 patients in their overall analysis sample, crude OR values comparing use in patients aged 85–99 years with those aged 15–64 years ranged notably from 0.02 (95% CI 0.01 to 0.02) in Scotland to 0.14 (95% CI 0.12 to 0.16) in Ontario, that is, the odds of chemotherapy use in the oldest compared with the youngest group were approximately 50-fold lower in Scotland and sevenfold lower in Ontario.

For non-advanced-stage (stages 1–3 or L-R), age gradients were similar across jurisdictions, with Norway, New South Wales and Ontario having higher than average use across all age groups.

For advanced stage (stage 4 or D), Norway, New South Wales and Ontario had higher than average use of chemotherapy in younger patients, whereas all jurisdictions except Ontario had low use of chemotherapy for patients aged 85–99 years, which was slightly higher than average in Norway, Alberta, British Columbia and New South Wales. In Ontario, while use of chemotherapy decreased with increasing age, use in patients aged 85–99 years was substantially higher than elsewhere (17.5%, compared with up to 6.6% in other jurisdictions).

### Radiotherapy use by age and stage

Interjurisdictional patterns of radiotherapy use by age were more variable than those observed for chemotherapy (figure 2B). Among jurisdictions with over 10 000 patients in their overall analysis sample, crude OR values comparing use in patients aged 85–99 years with those aged 15–64 ranged from 0.18 (95% CI 0.15 to 0.20) in Scotland to 0.93 (95% CI 0.85 to 1.02) in Ontario, that is, the odds of radiotherapy use in the oldest compared with the youngest group varied from sixfold lower to minimal or no difference, respectively. These observations can be further elucidated when considering age group and stage categories jointly, where three radiotherapy use patterns can be distinguished:

- Pattern A: in England, Northern Ireland, Scotland, Wales and Saskatchewan-Manitoba, radiotherapy use decreased with increasing age (particularly steeply for advanced-stage cancer) following a monotonic pattern.
- Pattern B: in Alberta, Atlantic Canada, British Columbia, New South Wales and Norway, in non-advanced stage, age differences in radiotherapy use were less pronounced than in pattern A jurisdictions, whereas in advanced stage, there were steep decreases in use with increasing age, similar to pattern A jurisdictions.
- Pattern C: uniquely in Ontario, radiotherapy use increased with increasing age in non-advanced stage, while it decreased with increasing age in advanced stage, although less steeply than in pattern A jurisdictions. Radiotherapy use for patients diagnosed in advanced stage in Ontario was the lowest among other jurisdictions for the youngest patients, while it was the highest for the oldest.

### Treatment use by sex and stage

**Figure 3:**
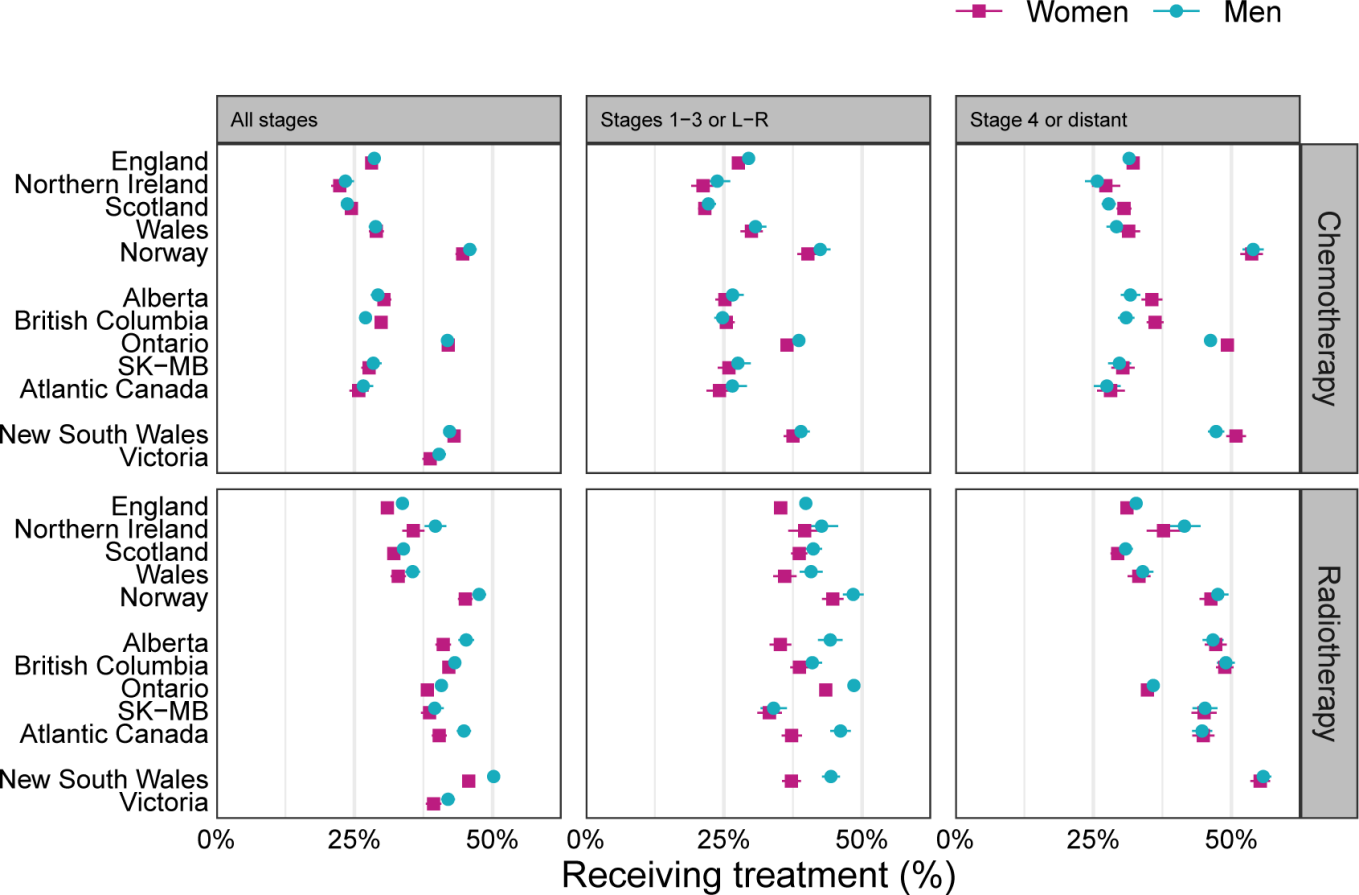
Proportion of patients treated with chemotherapy (top) and radiotherapy (bottom), by sex. Squares or circles show the point estimate, and horizontal lines show 95% CIs. L, localised; R, regional; SK-MB, Saskatchewan-Manitoba. See also online supplemental appendix 2 table 3.

Chemotherapy use was similar in women and men overall (32% in both, figure 3), but was slightly lower in women than men in non-advanced stage (29% in women vs 31% in men), and vice versa in advanced stage (36% in women vs 35% in men); these small differences varied by jurisdiction. For example, in patients diagnosed in advanced stage, Ontario and New South Wales had higher than average chemotherapy use overall, higher in women than men; Alberta and British Columbia had lower than average use overall, higher in women than men; Norway had higher than average use overall, with minimal differences by sex and the other jurisdictions had lower than average use overall, and small/minimal differences by sex.

Radiotherapy use was lower in women than men (35% in women vs 38% in men). This was chiefly due to differences in radiotherapy use in non-advanced stage (38% in women vs 42% in men), as radiotherapy use in advanced stage was only slightly higher in men (36% in women, 37% in men). Sex differences in radiotherapy use were particularly large for patients with stages 1–3 or L-R cancer in Alberta, Atlantic Canada and New South Wales.

### Time to treatment

**Figure 4:**
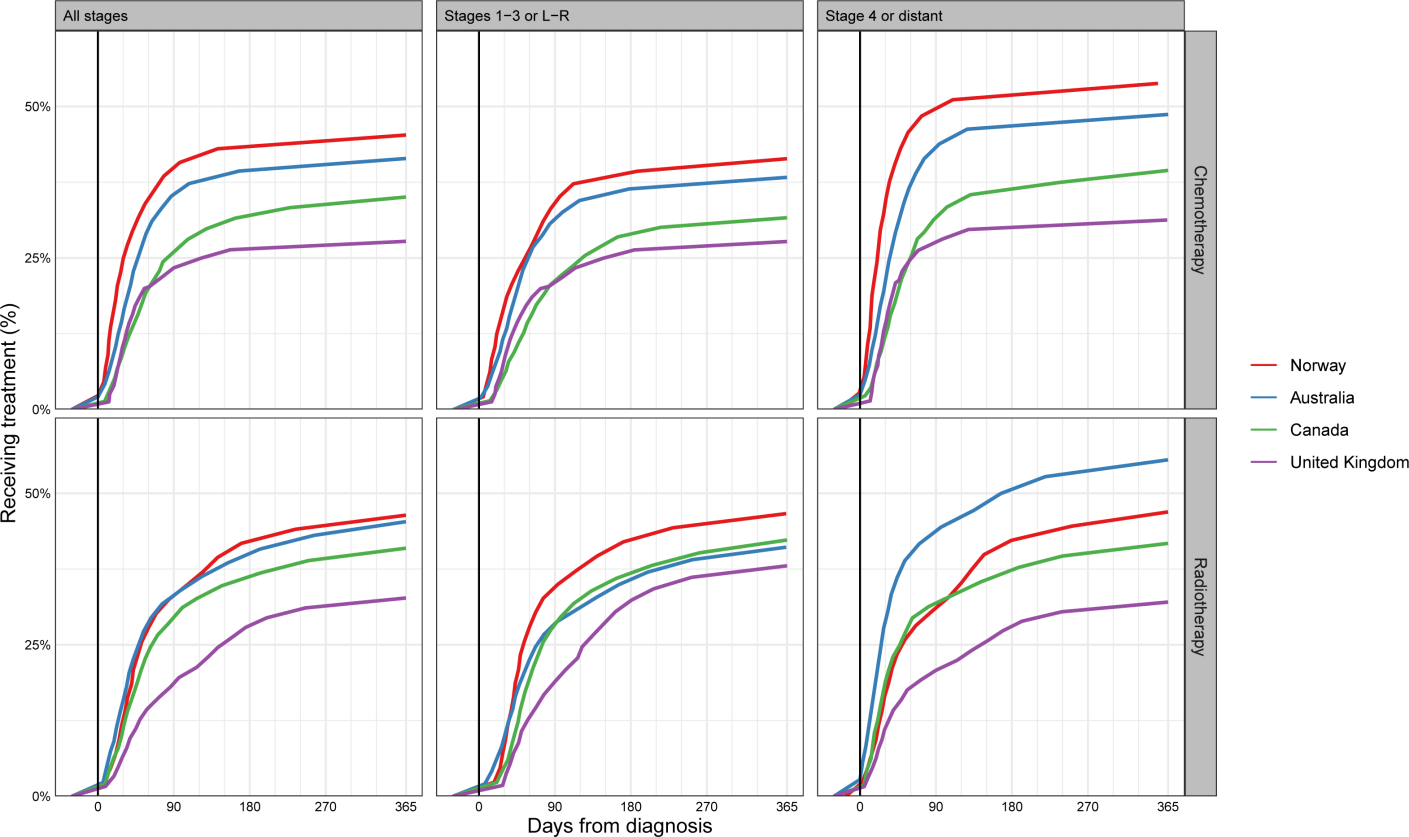
Cumulative percentage of patients in each country who had received chemotherapy (top) or radiotherapy (bottom) treatment, by elapsed time since diagnosis, for all stages, stages 1–3 (or localised (L)/regional (R)) or stage 4 (or distant). Stage-specific results for Australia are based on New South Wales data only. See also online supplemental appendix 2 tables 4 and 5.

**Figure 5:**
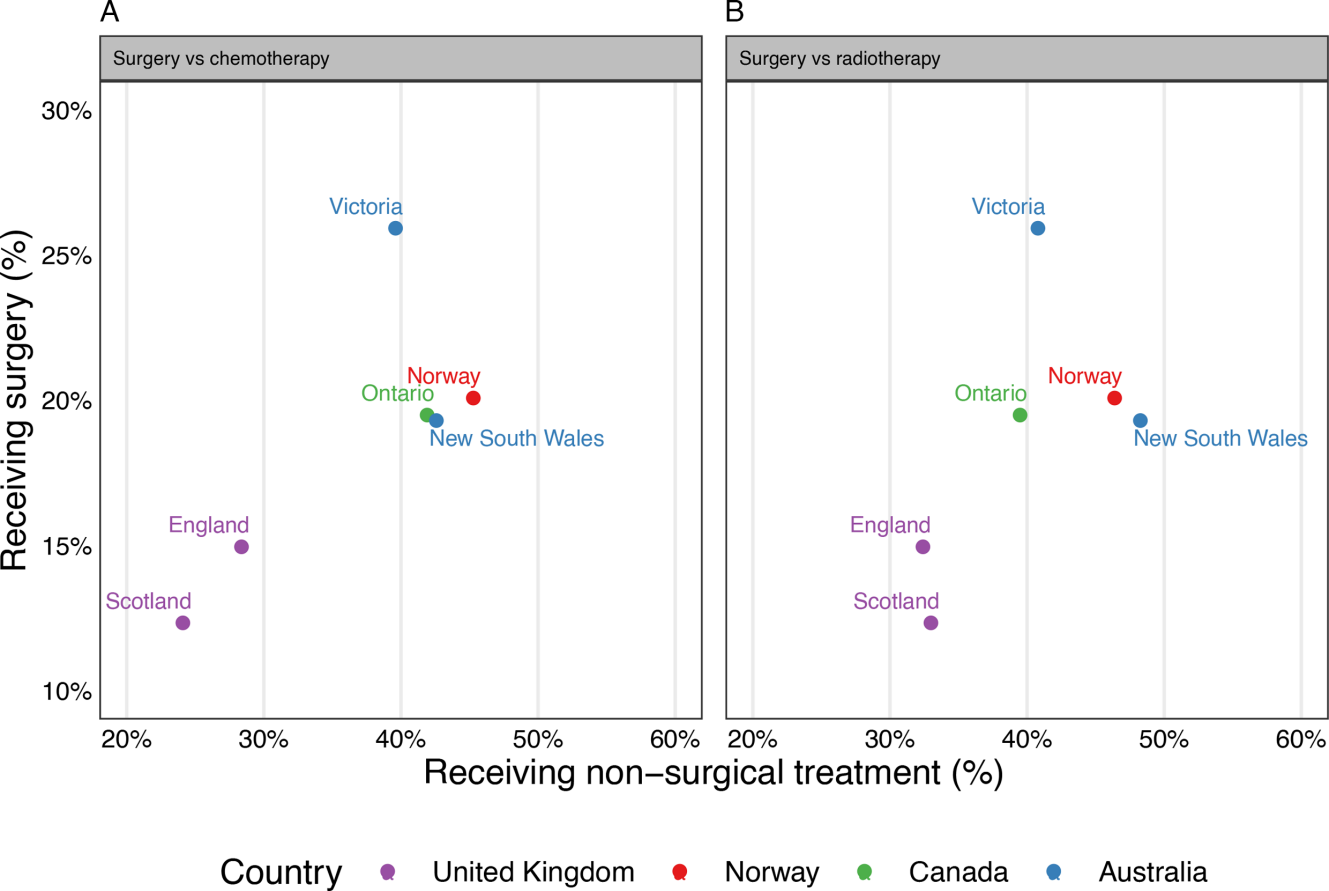
(A—left, B—right) Scatter plot of jurisdictional use of surgery versus chemotherapy and surgery versus radiotherapy for a subset of jurisdictions. Coloured circles show jurisdiction-specific use, all patients with lung cancer, with jurisdictions within the same country having the same colour circle. Data related to surgery use do not always align with study period for chemotherapy or radiotherapy use, or population basis (see ‘Methods’ section). Pearson’s r for surgery versus chemotherapy=0.75 (95% CI to 0.15, 0.97); Pearson’s r for surgery versus radiotherapy=0.60 (95% CI to 0.42, 0.95).

**Figure 6:**
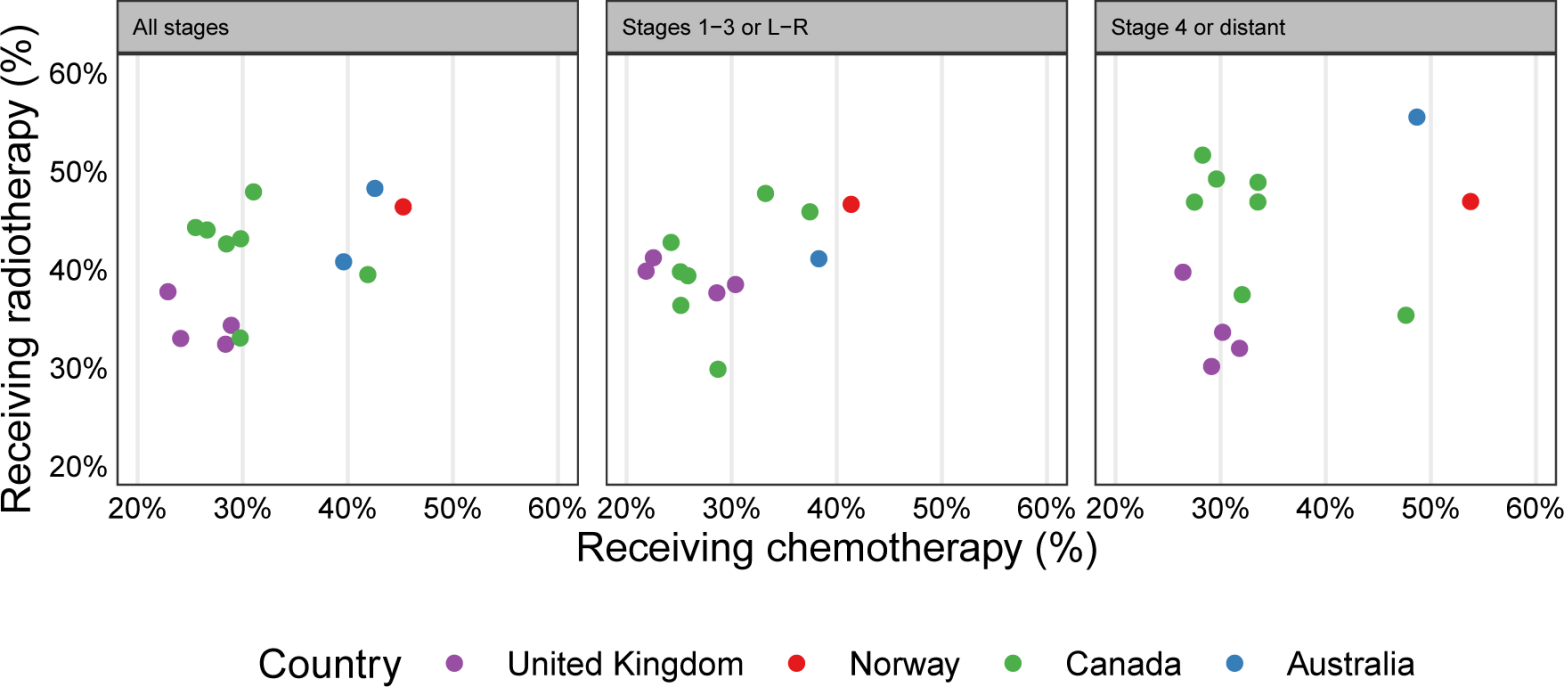
Scatter plot of jurisdictional use of radiotherapy versus chemotherapy, for all stages, stages 1–3 (or localised (L)/regional (R)), or stage 4 (or distant). Coloured circles show jurisdiction-specific use, with jurisdictions within the same country having the same colour circle.

Time to treatment initiation varied substantially between jurisdictions (figure 4, online supplemental appendix 1 figure 3–6). For example, in the UK, fewer than 15% of patients received chemotherapy within 30 days of diagnosis, compared with >22% of patients in Norway. In general, for both chemotherapy and radiotherapy, the jurisdictions that treated a higher proportion of patients also had shorter times to treatment initiation (ie, ‘higher providers were also faster providers’). The cumulative percentage of treated patients grew rapidly soon after the diagnosis, with generally only a few patients starting relatively late (eg, 180 days or longer) after their diagnosis, for either treatment.

### Ecological comparisons of jurisdictional-level use of chemotherapy, radiotherapy and surgery

In six jurisdictions for which we could source jurisdiction-level data on surgery use during relevant study periods, we observed that jurisdictions where more patients received chemotherapy or radiotherapy were generally also those where more patients received surgery (figure 5). While correlations were not statistically significant, there was no indication of lower use of surgery in jurisdictions with higher use of chemotherapy or radiotherapy.

When considering the use of chemotherapy alongside the use of radiotherapy across the jurisdictions (figure 6), we can see a group of high-chemotherapy/high-radiotherapy jurisdictions (Norway, Ontario, New South Wales, Victoria), a group of average-chemotherapy/high-radiotherapy jurisdictions (most other Canadian jurisdictions) and a group of average-chemotherapy/low-radiotherapy jurisdictions (UK, Saskatchewan). Differences in chemotherapy use were similar in both advanced and non-advanced stage groups, while differences in radiotherapy use appeared more pronounced in stage 4 or distant.

**Figure 7:**
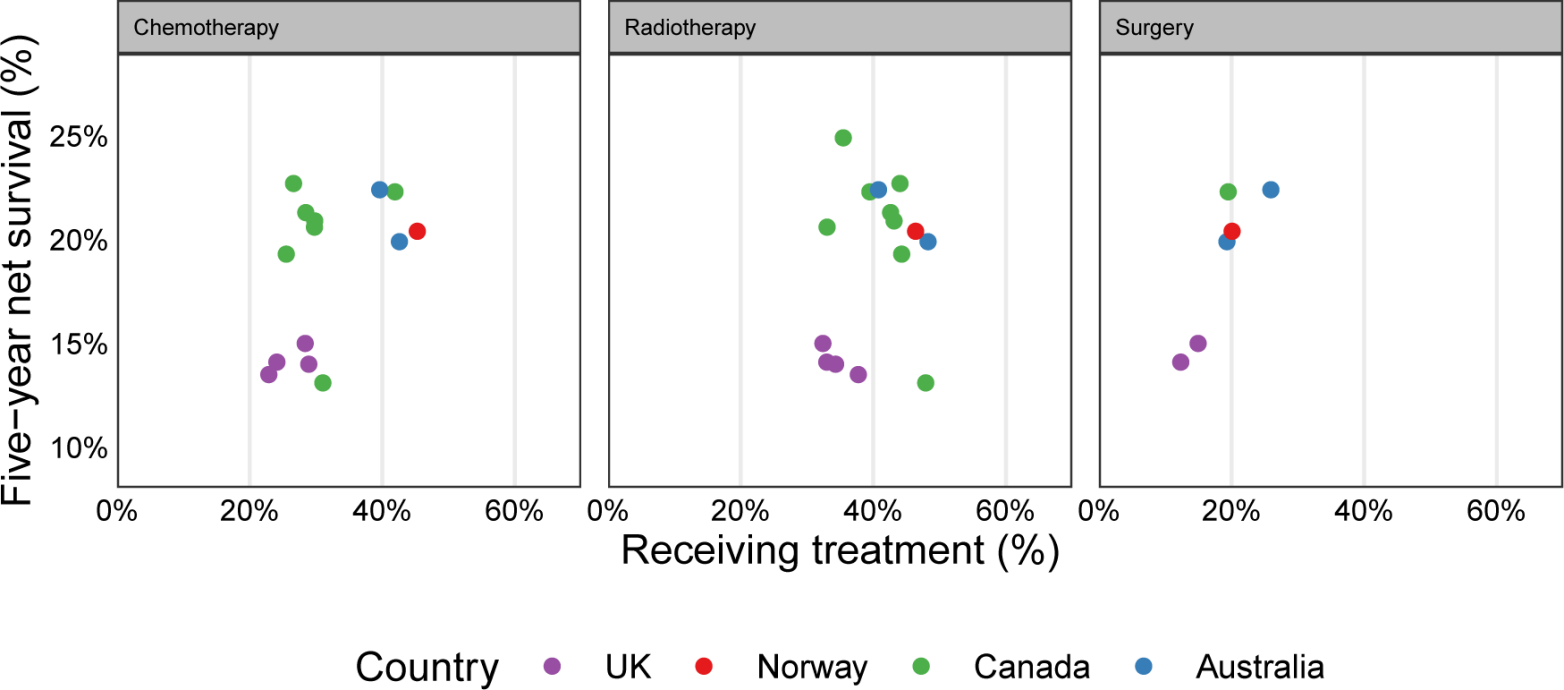
Scatter plot of jurisdictional 5-year net survival against use of chemotherapy, radiotherapy and surgery. Data for 5-year net survival from Arnold *et al*^1^; data on chemotherapy and radiotherapy use from the study analysis samples; for data on surgery use, please see ‘Methods’ section. Same colour is used for estimates of jurisdictions of the same country.

Exploratory comparisons of previously reported 3-year and 5-year net survival^1 2^ against jurisdictional use of chemotherapy, radiotherapy and, where possible based on existing literature,^6^,^11^ surgery found very weak evidence (p<0.1) of associations between higher use of chemotherapy in all patients, or higher use of radiotherapy in patients with metastatic cancer, and higher survival. There was weak evidence (p=0.04) of an association between higher use of surgery and higher survival in all patients (figure 7, stage-specific comparisons shown in online supplemental appendix 1 figure 7). Typically, correlations were not statistically significant (online supplemental appendix 1 table 3), and results must be interpreted with care.

## Discussion

There was substantial variability in the use of chemotherapy and radiotherapy for patients with lung cancer across the studied jurisdictions. While the use of chemotherapy decreased particularly sharply with increasing age within each jurisdiction, patterns of radiotherapy use by age varied substantially between jurisdictions, with Ontario notably different from elsewhere. Differences by sex, and for specific sex-stage combinations, were generally small, with the exception being radiotherapy use in patients with stage 1–3 or L-R lung cancer in Alberta, Atlantic Canada and New South Wales—where women were considerably less likely to receive radiotherapy than men.

Exploratory ecological analyses produced weak suggestions of higher survival in jurisdictions that had higher treatment use. These associations must be interpreted in the context of the small number of jurisdictions and the weaknesses of all ecological analyses; it would not be safe to conclude that increasing chemotherapy utilisation in a jurisdiction would necessarily increase survival. However, it seems reasonable to conclude that increasing the proportion of patients receiving appropriate, guideline-concordant, treatment will improve outcomes.

The findings indicate that the propensity of different health systems to use chemotherapy or radiotherapy in patients with lung cancer varies substantially, and stage-specific analyses suggest that factors other than stage at diagnosis are likely to be implicated. Considering the findings overall, Norway, Ontario and New South Wales tended to have higher than average use of both chemotherapy and radiotherapy, overall and for most age/sex/stage strata; the four UK nations tended to have lower than average use, both overall and for most age/sex/stage strata; with the other jurisdictions falling between these two patterns.

### Comparisons with prior literature

There is little international evidence on treatment use for lung cancer, with previous studies only covering a small number of jurisdictions.^819^,^21^ No other studies to date included a comparable number of jurisdictions and the use of both chemotherapy and radiotherapy, except our earlier meta-analyses.^3 4^ In the present study, we present additional details on the treatment of lung cancer, including by stage, age, sex, age-stage and sex-stage strata, and jointly consider both use and time-to-treatment.

Some sex differences were observed in the use of either treatment, particularly for radiotherapy for stages 1–3 or L-R disease, with greater observed use in men. Two factors may be contributing to this difference. First, in ICBP jurisdictions during the study era, compared with women, men were somewhat more likely to be diagnosed with non-small cell lung cancer,^22^ for which radiotherapy is generally more frequently indicated compared with small cell lung cancer (online supplemental appendix 1 table 3). Second, the higher use of radiotherapy in men with non-advanced disease compared with women may reflect a greater burden of comorbidity in men, making use of radiotherapy instead of lung excision surgery more suitable in the context of greater average operative risk in men.

### Strengths and limitations

The key strength of this international study is the use of large and harmonised population-based datasets. Thus, the definitions of the measured treatment outcomes and of the age, sex and stage strata are as similar as possible between different jurisdictions, and data come from similar study periods. Our results plausibly describe chemotherapy and radiotherapy treatment practice in each jurisdiction during the mid-2010s. To our knowledge, we provide the first detailed summaries of time from diagnosis to initiation of chemotherapy or radiotherapy across different countries.

The study has limitations. Although we have captured the use of chemotherapy and radiotherapy, we only had indirect information on the use of surgery in most jurisdictions. Surgery is used for early stage disease; theoretically, jurisdictions with lower use of radiotherapy for early stage disease might have had higher use of surgery. However, our correlational analysis of jurisdiction-level use of surgery and radiotherapy does not lend obvious support to such a hypothesis (figure 5); in fact, we observe that jurisdictions with higher reported surgery use also had higher use of radiotherapy, although the correlation was not statistically significant.

We could not directly assess whether variation in treatment was warranted and did not try to establish whether treatment was in line with treatment guidelines of the era.^23^,^25^ We did not examine morphological subtype, but previous evidence suggests that any jurisdictional differences are likely to be small (online supplemental appendix 1 table 4).^22^ Patients’ comorbidities, performance status and other aspects of their health influence treatment options but are challenging to measure consistently.^26^ While such factors may impact overall comparisons of treatment use, we believe age-specific comparisons will be far less affected. Comorbidities and frailty are likely to be less of an issue in younger age groups. The lack of frailty information is a limitation when comparing older populations; while we do not expect large differences in the frailty of patients with lung cancer between jurisdictions, we would have preferred to examine this directly. We also did not have information on treatment intent. Specifically, radiotherapy may be administered as radical treatment in early stage disease, or for palliation in advanced stage for symptom control. Stage-specific analysis suggests that there was more pronounced variation in use of palliative radiotherapy, as the jurisdictional variance in radiotherapy use appeared larger for stage 4 disease than stages 1–3.

Stage information was highly complete (except for Victoria), but within jurisdictions, between 1% and 15% of patients had missing stage information, which may introduce bias into stage-specific treatment comparisons. Given the overall high completeness of stage information and the observed proportion of patients without stage information receiving treatment, we consider the potential for bias in stage-specific treatment estimates from missing stage information to be limited (see online supplemental appendix 3 section 1).

Data relate to the mid-2010s, and care must be taken when extrapolating to the present day. Very substantial changes in clinical practice have occurred since the study period, including the advent of immunotherapies and targeted treatments, greater use of stereotactic radiotherapy in comorbid patients and greater use of surgical intervention with neo-adjuvant/adjuvant treatments (online supplemental appendix 3 section 2). The pattern of greater use of radiotherapy with increasing age in patients with non-advanced disease in Ontario is an early sign of such a change, suggestive of early adoption of stereotactic radiotherapy—concordant with other evidence.^27^ Additional factors that limit generalisability over time are changes in the incident sex ratio (and possible related differences in morphological case-mix, online supplemental appendix 1 table 4) and the progressive introduction of lung cancer screening. With that said, treatment data from England up to 2021,^28^ and Norway up to 2023,^6 29^ suggest changes in use of the three broad categories of treatment have been gradual (ignoring changes in the exact type of chemotherapy or radiotherapy employed). While lung cancer treatment has evolved, we believe our results provide an indication of the intensity of current management of lung cancer.

### Implications

For most of the age/sex/stage strata and overall, jurisdictions previously reported to have high survival (eg, Norway, Ontario and New South Wales), tended to have higher than average use of both chemotherapy and radiotherapy. For most age/sex/stage strata and overall, the four UK jurisdictions tended to have lower-than-average treatment use and longer-than-average time to treatment initiation for both treatments. The same pattern was observed in Saskatchewan, and although only radiotherapy data were available, in New Brunswick. Some studies have linked delays in treatment initiation with worse outcomes.^30 31^ For the UK jurisdictions, prior evidence has also shown lower survival rates.

Directly examining the use of surgery in different jurisdictions is an obvious next step, but our findings amplify previous evidence suggesting that variation in management is one of the likely drivers of international differences in lung cancer survival.^22 32^ However, international variation also exists in the percentage of patients diagnosed following an emergency hospital admission, a diagnostic route associated with adverse prognosis.^33 34^ Efforts to promote more timely diagnosis are therefore warranted to improve survival, and the introduction of lung cancer screening offers great potential to shift diagnoses towards non-advanced stages.

Some patients do not stand to benefit from treatment, either due to advanced cancer or due to poor general health. Indeed, one possible mechanism for lower use of chemotherapy or radiotherapy in older patients is that they may be experiencing higher mortality than younger patients soon after the diagnosis, either due to their cancer or iatrogenic complications from its initial management, or due to other competing causes, precluding receipt of the examined treatments.^33^,^35^ Our focus in this study has largely been on the likelihood of undertreatment in some jurisdictions, but there may also be problems with futile treatment (from which no overall clinical benefit is derived), perhaps especially in jurisdictions that treated the highest proportion of their patients. Future work should aim to examine variation in guideline-concordant management to allow assessment of both undertreatment and overtreatment.

## Conclusion

Use of chemotherapy and radiotherapy in lung cancer, and variation in their use by age, differs substantially between jurisdictions, and such differences transcend disease stage categories. Although our study period relates to the past decade, reasons for these patterns of variable use and time to chemotherapy and radiotherapy need to be explored in more detail as they are likely to have contributed to persistent interjurisdictional variation in survival.

## Supplementary material

10.1136/bmjonc-2025-000800 online supplemental file 1

10.1136/bmjonc-2025-000800 online supplemental file 2

10.1136/bmjonc-2025-000800 online supplemental file 3

## Data Availability

Data may be obtained from a third party and are not publicly available.
