# Supplementary material for: Chemotherapy and radiotherapy use in patients with lung cancer in Australia, Canada, the UK and Norway 2012–2017: an ICBP population-based study
Source: BMJ Oncol. 2025 Jul 11;4(1):e000800. doi: 10.1136/bmjonc-2025-000800 (PMC12258358; doi:10.1136/bmjonc-2025-000800)
Supplement: online supplemental file 1 [file bmjonc-4-1-s001.pdf]

APPENDIX 1. ADDITIONAL TREATMENT PLOTS AND TABLES.

Contents

Appendix 1 Figure 1. Change in five-year net survival from 1995-1999 to 2010-2014 for lung cancer in countries in the International Cancer Benchmarking Partnership. 2

Appendix 1 Table 1. Analysis samples by diagnosis year and jurisdiction. 3

Appendix 1 Table 2. Analysis illustrating the likely impact of not capturing oral chemotherapy when used as monotherapy during the study period, by age group. 4

Appendix 1 Figure 2. Proportion of lung cancer patients in each jurisdiction who received chemotherapy (top) or radiotherapy (bottom) treatment, by stage at diagnosis. 5

Appendix 1 Figure 3. Cumulative percentage of treated patients by elapsed time since diagnosis, for Canadian jurisdictions. 6

Appendix 1 Figure 4. Cumulative percentage of treated patients by elapsed time since diagnosis, for Australian jurisdictions. 7

Appendix 1 Figure 5. Cumulative percentage of treated patients by elapsed time since diagnosis, for UK jurisdictions. 8

Appendix 1 Figure 6. Cumulative percentage of treated patients by elapsed time since diagnosis, for Norway. 9

Appendix 1 Figure 7. Stage-specific scatter plot of jurisdictional three-year net survival for NSCLC against use of chemotherapy and radiotherapy for any lung cancer. 10

Appendix 1 Table 3. Pearson’s correlation coefficients for pairwise comparisons of treatment use and net survival, both overall (five-years) and stage-specific (three-years). 11

Appendix 1 Table 4. Lung cancer tumour types in ICBP jurisdictions. 12

Bibliography for Appendix 1 13

Appendix 1 Figure 1. Change in five-year net survival from 1995-1999 to 2010-2014 for lung cancer in countries in the International Cancer Benchmarking Partnership.

Data from Arnold et al [1].
Infographic produced by Cancer Research UK. Available at https://www.cancerresearchuk.org/health-professional/data-and-statistics/international-cancer-benchmarking-partnership-icbp/resources

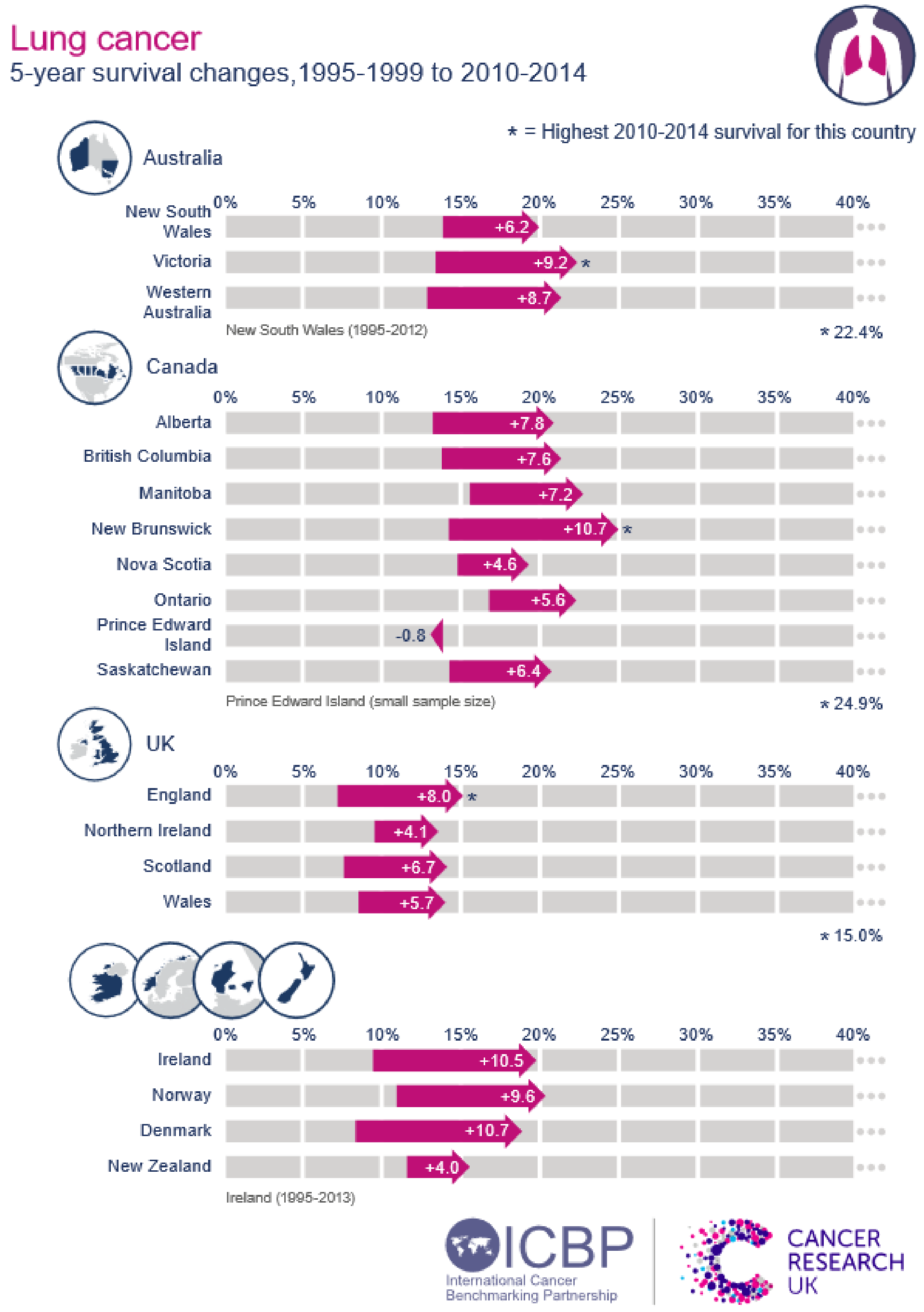

Appendix 1 Table 1. Analysis samples by diagnosis year and jurisdiction.

Y: Data provided.

Chemotherapy

	2012	2013	2014	2015	2016	2017
Norway		Y	Y	Y	Y	
England		Y	Y	Y	Y	
Northern Ireland	Y	Y	Y	Y	Y	
Scotland			Y	Y	Y	Y
Wales		Y	Y	Y	Y	
Alberta	Y	Y	Y	Y	Y	
British Columbia	Y	Y	Y	Y	Y	
Nova Scotia	Y	Y	Y	Y	Y	
Manitoba	Y	Y	Y	Y	Y	
Ontario	Y	Y	Y	Y	Y	
Prince Edward Island	Y	Y	Y	Y	Y	
Saskatchewan	Y	Y	Y	Y	Y	
New South Wales	Y	Y	Y	Y	Y	
Victoria	Y	Y	Y	Y	Y	

Radiotherapy

	2012	2013	2014	2015	2016	2017
Norway		Y	Y	Y	Y	
England		Y	Y	Y	Y	
Northern Ireland	Y	Y	Y		Y	
Scotland			Y	Y	Y	Y
Wales		Y	Y	Y	Y	
Alberta	Y	Y	Y	Y	Y	
British Columbia	Y	Y	Y	Y	Y	
New Brunswick	Y	Y	Y	Y	Y	
Newfoundland & Labrador	Y	Y	Y	Y	Y	
Nova Scotia	Y	Y	Y	Y	Y	
Manitoba	Y	Y	Y	Y	Y	
Ontario	Y	Y	Y	Y	Y	
Prince Edward Island	Y	Y	Y	Y	Y	
Saskatchewan	Y	Y	Y	Y	Y	
New South Wales	Y	Y	Y	Y	Y	
Victoria	Y	Y	Y	Y	Y	

Appendix 1 Table 2. Analysis illustrating the likely impact of not capturing oral chemotherapy when used as monotherapy during the study period, by age group.

The findings suggest that in a scenario where oral chemotherapy was used as monotherapy and oral monotherapy was not captured in data sources, the potential under-counting from this mechanism is relatively small. Data from England, New South Wales, and Scotland, where information on oral chemotherapy was being collected during the study period. Data reproduced from McPhail et al.[2]

	England		New South Wales		Scotland	
**Age group**	**All chemotherapy**	**Oral only chemotherapy**	**All chemotherapy**	**Oral only chemotherapy**	**All chemotherapy**	**Oral only chemotherapy**
15-64	48·5%	2·2%	60·8%	3·9%	48·1%	1·8%
65-74	36·0%	1·7%	49·1%	3·0%	29·9%	1·2%
75-84	16·5%	1·4%	26·8%	3·2%	11·1%	0·7%
85-99	2·3%	0·7%	8·3%	2·9%	1·6%	0·6%

Appendix 1 Figure 2. Proportion of lung cancer patients in each jurisdiction who received chemotherapy (top) or radiotherapy (bottom) treatment, by stage at diagnosis.

Hollow circles show results for Norway and New South Wales, where summary stage was recorded rather than TNM, with Localised shown in Stage 1, Regional shown in Stage 3 and Distant shown in Stage 4. Due to imperfect alignment between these stage definitions, meta-analyses in this figure did not include Norway or New South Wales.

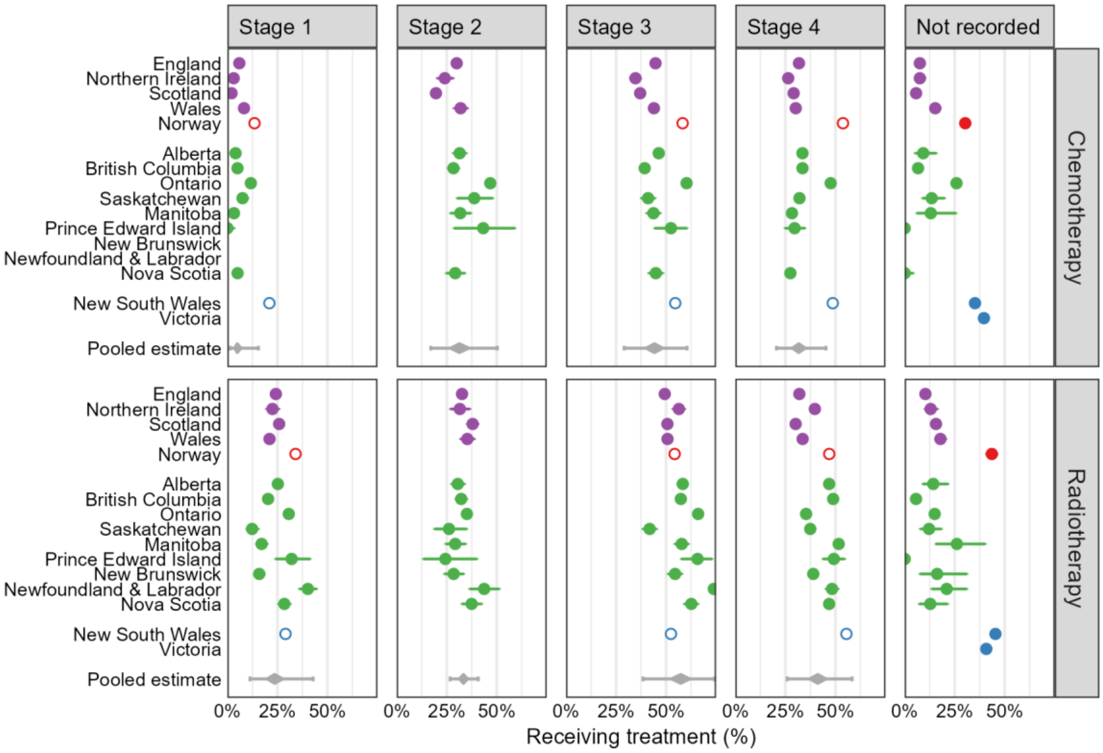

Appendix 1 Figure 3. Cumulative percentage of treated patients by elapsed time since diagnosis, for Canadian jurisdictions.

Grey lines show results for jurisdictions in other ICBP countries.

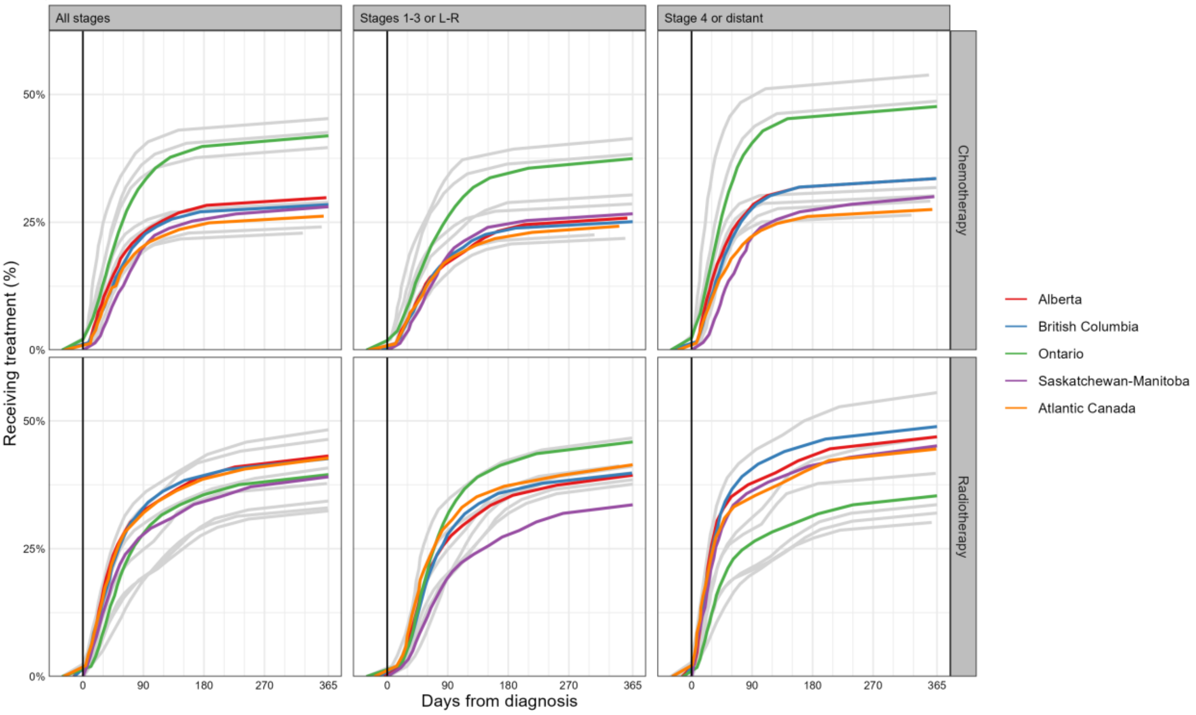

Appendix 1 Figure 4. Cumulative percentage of treated patients by elapsed time since diagnosis, for Australian jurisdictions.

Grey lines show results for jurisdictions in other ICBP countries.

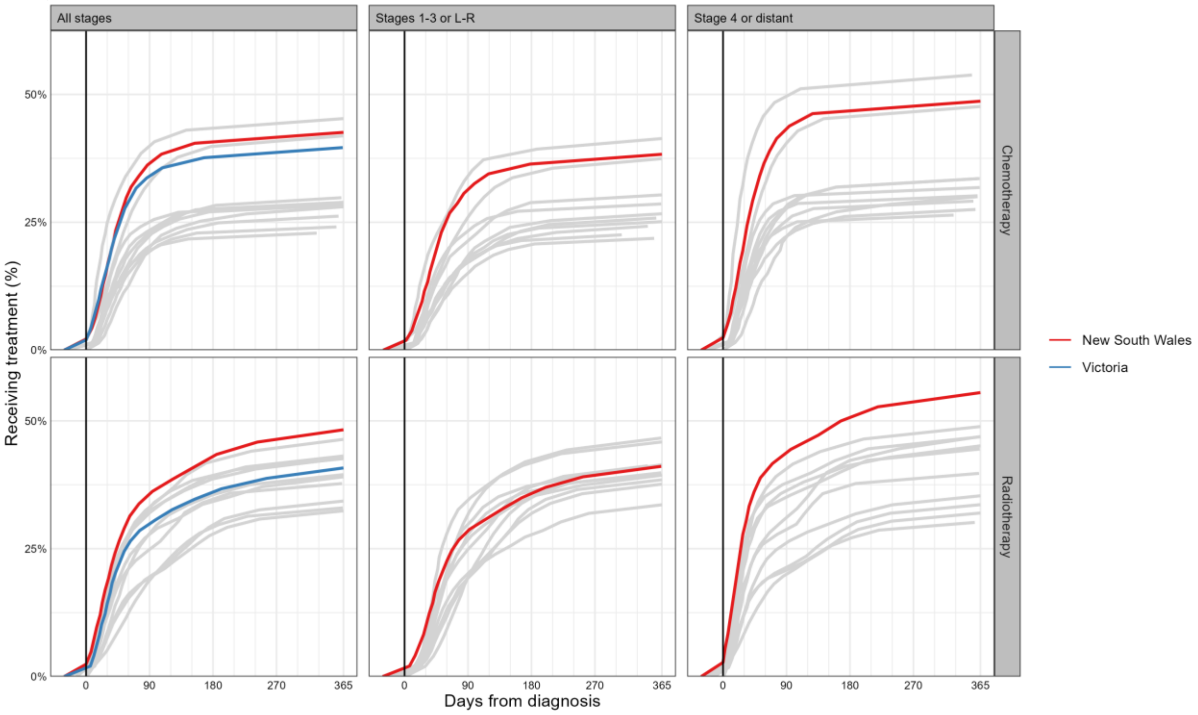

Appendix 1 Figure 5. Cumulative percentage of treated patients by elapsed time since diagnosis, for UK jurisdictions.

Grey lines show results for jurisdictions in other ICBP countries.

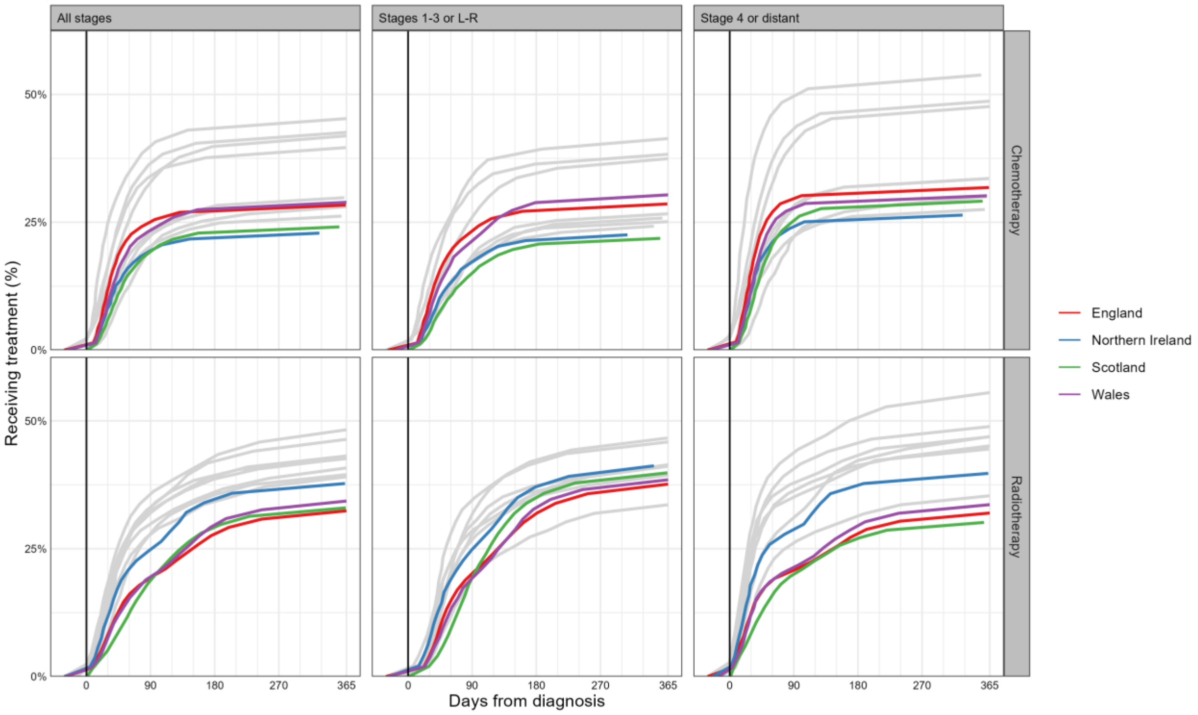

Appendix 1 Figure 6. Cumulative percentage of treated patients by elapsed time since diagnosis, for Norway.

Grey lines show results for jurisdictions in other ICBP countries.

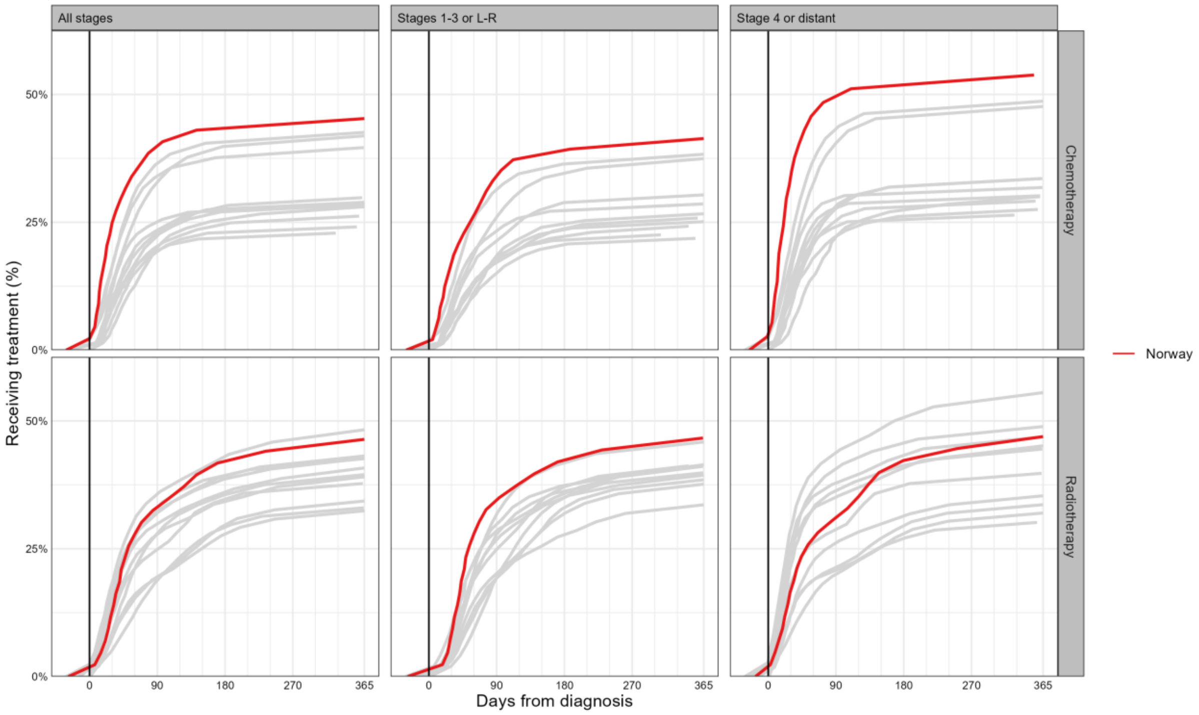

Appendix 1 Figure 7. Stage-specific scatter plot of jurisdictional three-year net survival for NSCLC against use of chemotherapy and radiotherapy for any lung cancer.

Net survival data from Araghi et al.[3]

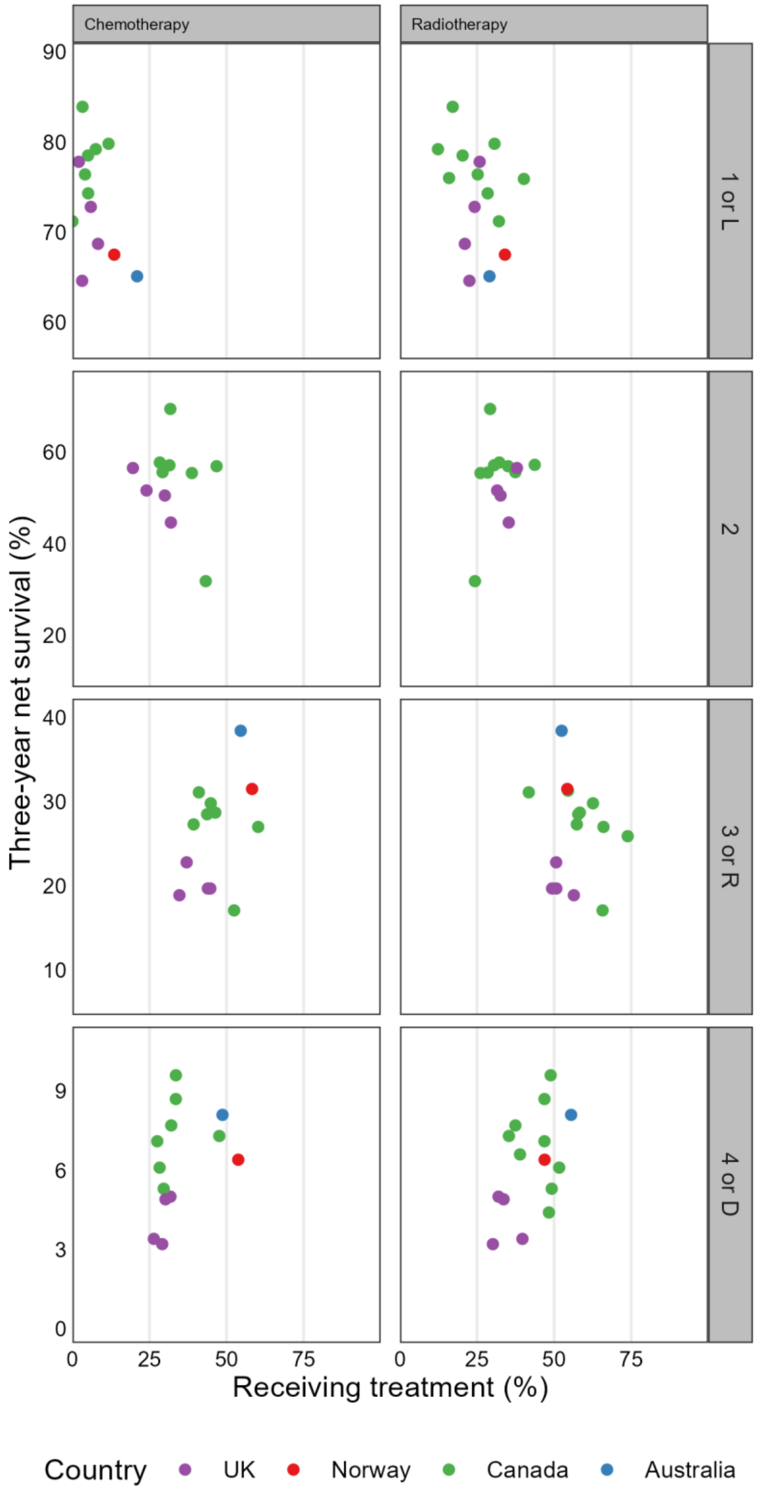

Appendix 1 Table 3. Pearson's correlation coefficients for pairwise comparisons of treatment use and net survival, both overall (five-years) and stage-specific (three-years).

This analysis is not adequately powered to detect reasonable correlations; any statistically significant correlations are likely to be overestimates, while failure to detect a correlation does not allow us to draw conclusions about the likely direction or strength of the true correlation. 5-year survival (all-stage) estimates from Arnold et al. [1]; 3-year (stage-specific) survival estimates from Araghi et al.[3].

Comparison	Stage	Survival follow-up (years)	Pearson's R	(95% CI)	p
Chemotherapy vs radiotherapy	All stages		0.44	(-0.12, 0.79)	0.114
Chemotherapy vs radiotherapy	Stages 1-3 or L-R		0.45	(-0.17, 0.81)	0.145
Chemotherapy vs radiotherapy	Stage 4 or distant		0.29	(-0.34, 0.74)	0.360
Net survival vs chemotherapy	All stages	5	0.49	(-0.06, 0.81)	0.078
Net survival vs chemotherapy	Stage 1 or L	3	-0.38	(-0.77, 0.21)	0.195
Net survival vs chemotherapy	Stage 2	3	-0.30	(-0.76, 0.37)	0.370
Net survival vs chemotherapy	Stage 3 or R	3	0.37	(-0.23, 0.76)	0.217
Net survival vs chemotherapy	Stage 4 or D	3	0.38	(-0.21, 0.77)	0.196
Net survival vs radiotherapy	All stages	5	0.22	(-0.33, 0.66)	0.436
Net survival vs radiotherapy	Stage 1 or L	3	-0.32	(-0.71, 0.24)	0.253
Net survival vs radiotherapy	Stage 2	3	0.28	(-0.33, 0.72)	0.363
Net survival vs radiotherapy	Stage 3 or R	3	-0.14	(-0.61, 0.40)	0.619
Net survival vs radiotherapy	Stage 4 or D	3	0.46	(-0.06, 0.79)	0.081
Net survival vs surgery	All stages	5	0.84	(0.08, 0.98)	0.038

Appendix 1 Table 4. Lung cancer tumour types in ICBP jurisdictions.

Data calculated from Morgan et al.[4]

		Men		Women	
**Australia**	**SCLC**	2,199	10.7%	1,626	11.4%
	**NSCLC**	15,468	75.4%	10,661	74.8%
	**Other**	53	0.3%	29	0.2%
	**Unspecified**	2,800	13.6%	1,930	13.5%
	**Total**	20,520		14,246	
**UK**	**SCLC**	12,599	10.8%	12,154	12.4%
	**NSCLC**	72,303	61.8%	55,215	56.3%
	**Other**	281	0.2%	213	0.2%
	**Unspecified**	31,779	27.2%	30,532	31.1%
	**Total**	11,6962		98,114	
**Canada**	**SCLC**	5,678	10.8%	5,632	11.7%
	**NSCLC**	36,600	69.5%	33,247	69.2%
	**Other**	82	0.2%	63	0.1%
	**Unspecified**	10,280	19.5%	9,079	18.9%
	**Total**	52,640		48,021	

Bibliography for Appendix 1

1 Arnold M, Rutherford MJ, Bardot A, Ferlay J, Andersson TM-L, Myklebust TÅ, Tervonen H, Thursfield V, Ransom D, Shack L, Woods RR, Turner D, Leonfellner S, Ryan S, Saint-Jacques N, De P, McClure C, Ramanakumar AV, Stuart-Panko H, Engholm G, Walsh PM, Jackson C, Vernon S, Morgan E, Gavin A, Morrison DS, Huws DW, Porter G, Butler J, Bryant H, Currow DC, Hiom S, Parkin DM, Sasieni P, Lambert PC, Møller B, Soerjomataram I, Bray F. Progress in cancer survival, mortality, and incidence in seven high-income countries 1995–2014 (ICBP SURVMARK-2): a population-based study. *The Lancet Oncology*. 2019;20:1493–505. doi: 10.1016/S1470-2045(19)30456-5

2 McPhail S, Barclay ME, Johnson SA, Swann R, Alvi R, Barisic A, Bucher O, Creighton N, Denny CA, Dewar RA, Donnelly DW, Dowden JJ, Downie L, Finn N, Gavin AT, Habbous S, Huws DW, May L, McClure CA, Møller B, Musto G, Nilssen Y, Saint-Jacques N, Sarker S, Shack L, Tian X, Thomas RJS, Thomson CS, Wang H, Woods RR, You H, Lyratzopoulos G, Altman AD, Bennett D, Butler J, Cameron DA, Crosby T, Davies L, Dixon E, Filsinger B, Forster K, Fung S, Navas EG, Guren MG, Han J, Hanna L, Harrison S, Lawler M, Little AL, Mala T, Merrett N, Morrison DS, Nelson G, Peacock SJ, Ransom DT, Ray-Coquard I, Warlow JL, Whitfield E, Zalcberg JR. Use of chemotherapy in patients with oesophageal, stomach, colon, rectal, liver, pancreatic, lung, and ovarian cancer: an International Cancer Benchmarking Partnership (ICBP) population-based study. *The Lancet Oncology*. 2024;25:338–51. doi: 10.1016/S1470-2045(24)00031-7

3 Araghi M, Fidler-Benaoudia M, Arnold M, Rutherford M, Bardot A, Ferlay J, Bucher O, De P, Engholm G, Gavin A, Kozie S, Little A, Møller B, St Jacques N, Tervonen H, Walsh P, Woods R, O'Connell DL, Baldwin D, Elwood M, Siesling S, Bray F, Soerjomataram I, ICBP SURVMARK-2 Local Leads, ICBP SURVMARK-2 Academic Reference Group. International differences in lung cancer survival by sex, histological type and stage at diagnosis: an ICBP SURVMARK-2 Study. *Thorax*. 2022;77:378–90. doi: 10.1136/thoraxjnl-2020-216555

4 Morgan E, Arnold M, Rutherford MJ, Bardot A, Ferlay J, De P, Engholm G, Jackson C, Little A, Saint-Jacques N, Walsh P, Woods RR, O'Connell DL, Bray F, Parkin DM, Soerjomataram I. The impact of reclassifying cancers of unspecified histology on international differences in survival for small cell and non-small cell lung cancer (ICBP SURVMARK -2 project). *Intl Journal of Cancer*. 2021;149:1013–20. doi: 10.1002/ijc.33620
