# Supplementary material for: Chemotherapy and radiotherapy use in patients with lung cancer in Australia, Canada, the UK and Norway 2012–2017: an ICBP population-based study
Source: BMJ Oncol. 2025 Jul 11;4(1):e000800. doi: 10.1136/bmjonc-2025-000800 (PMC12258358; doi:10.1136/bmjonc-2025-000800)
Supplement: online supplemental file 2 [file bmjonc-4-1-s002.pdf]

APPENDIX 2. DATA TABLES.

Contents

Appendix 2 Table 1a. Jurisdictional chemotherapy counts used to produce Figure 1 and Table 2. Counts between 1 and 9 are suppressed; where suppression has been applied, other counts are rounded to the nearest 10. 2

Appendix 2 Table 1b. Jurisdictional radiotherapy counts used to produce Figure 1 and Table 2. Counts between 1 and 9 are suppressed; where suppression has been applied, other counts are rounded to the nearest 10. 3

Appendix 2 Table 2a. Chemotherapy all stages age-specific jurisdictional treatment counts used to produce Figure 2. Counts between 1 and 9 are suppressed; where suppression has been applied, other counts are rounded to the nearest 10. For SK-MB, suppression was applied to the count for Manitoba. 5

Appendix 2 Table 2b. Chemotherapy stages 1-3 or L-R age-specific jurisdictional treatment counts used to produce Figure 2. Counts between 1 and 9 are suppressed; where suppression has been applied, other counts are rounded to the nearest 10. ... 6

Appendix 2 Table 2c. Chemotherapy stage 4 or distant age-specific jurisdictional treatment counts used to produce Figure 2. Counts between 1 and 9 are suppressed; where suppression has been applied, other counts are rounded to the nearest 10. For SK-MB, suppression was applied to the count for Saskatchewan. 7

Appendix 2 Table 2d. Radiotherapy all stages age-specific jurisdictional treatment counts used to produce Figure 2. 8

Appendix 2 Table 2e. Radiotherapy stages 1-3 or L-R age-specific jurisdictional treatment counts used to produce Figure 2. ... 9

Appendix 2 Table 2f. Radiotherapy stage 4 or distant age-specific jurisdictional treatment counts used to produce Figure 2.... 10

Appendix 2 Table 3a. Sex-specific jurisdictional chemotherapy counts used to produce Figure 3. 11

Appendix 2 Table 3b. Sex-specific jurisdictional radiotherapy counts used to produce Figure 3. 13

Appendix 2 Table 4. Key centiles of time-to-treatment aggregated to country-level, data relate to Figure 4. 15

Appendix 2 Table 5a. Key centiles of time-to-chemotherapy at jurisdiction-level, data relate to Appendix 1 Figures 3-6. 16

Appendix 2 Table 5b. Key centiles of time-to-radiotherapy at jurisdiction-level, data relate to Appendix 1 Figures 3-6. 17

Appendix 2 Table 1a. Jurisdictional chemotherapy counts used to produce Figure 1 and Table 2. Counts between 1 and 9 are suppressed; where suppression has been applied, other counts are rounded to the nearest 10.

				Received treatment		
**Country**	**Jurisdiction**	**Stage at diagnosis**	**Patients**	**N**	**%**	**(95% CI)**
United Kingdom	England	Stage 1	19,368	1,152	5.9%	(5.6%, 6.3%)
	England	Stage 2	9,742	2,920	30.0%	(29.1%, 30.9%)
	England	Stage 3	26,334	11,776	44.7%	(44.1%, 45.3%)
	England	Stage 4	68,141	21,667	31.8%	(31.4%, 32.1%)
	England	No recorded stage	11,811	895	7.6%	(7.1%, 8.1%)
	Northern Ireland	Stage 1	860	27	3.1%	(2.2%, 4.5%)
	Northern Ireland	Stage 2	437	105	24.0%	(20.3%, 28.2%)
	Northern Ireland	Stage 3	1,320	458	34.7%	(32.2%, 37.3%)
	Northern Ireland	Stage 4	2,594	685	26.4%	(24.7%, 28.1%)
	Northern Ireland	No recorded stage	541	41	7.6%	(5.6%, 10.1%)
	Scotland	Stage 1	2,781	57	2.0%	(1.6%, 2.6%)
	Scotland	Stage 2	1,249	245	19.6%	(17.5%, 21.9%)
	Scotland	Stage 3	3,809	1,411	37.0%	(35.5%, 38.6%)
	Scotland	Stage 4	9,245	2,693	29.1%	(28.2%, 30.1%)
	Scotland	No recorded stage	1,578	90	5.7%	(4.7%, 7.0%)
	Wales	Stage 1	1,326	110	8.3%	(6.9%, 9.9%)
	Wales	Stage 2	714	228	31.9%	(28.6%, 35.4%)
	Wales	Stage 3	2,074	911	43.9%	(41.8%, 46.1%)
	Wales	Stage 4	4,400	1,328	30.2%	(28.8%, 31.6%)
	Wales	No recorded stage	851	130	15.3%	(13.0%, 17.8%)
Norway	Norway	Localised	1,979	268	13.5%	(12.1%, 15.1%)
	Norway	Regional	3,259	1,899	58.3%	(56.6%, 60.0%)
	Norway	Distant	4,895	2,633	53.8%	(52.4%, 55.2%)
	Norway	No recorded stage	1,414	428	30.3%	(27.9%, 32.7%)
Canada	Alberta	Stage 1	1,765	72	4.1%	(3.3%, 5.1%)
	Alberta	Stage 2	747	235	31.5%	(28.2%, 34.9%)
	Alberta	Stage 3	1,666	772	46.3%	(44.0%, 48.7%)
	Alberta	Stage 4	5,111	1,714	33.5%	(32.3%, 34.8%)
	Alberta	No recorded stage	119	11	9.2%	(5.2%, 15.8%)
	British Columbia	Stage 1	2,298	116	5.0%	(4.2%, 6.0%)
	British Columbia	Stage 2	1,083	307	28.3%	(25.7%, 31.1%)
	British Columbia	Stage 3	3,005	1,181	39.3%	(37.6%, 41.1%)
	British Columbia	Stage 4	7,561	2,537	33.6%	(32.5%, 34.6%)
	British Columbia	No recorded stage	792	53	6.7%	(5.2%, 8.6%)
	Ontario	Stage 1	7,035	824	11.7%	(11.0%, 12.5%)
	Ontario	Stage 2	2,780	1,300	46.8%	(44.9%, 48.6%)
	Ontario	Stage 3	6,801	4,098	60.3%	(59.1%, 61.4%)
	Ontario	Stage 4	17,017	8,106	47.6%	(46.9%, 48.4%)
	Ontario	No recorded stage	1,459	378	25.9%	(23.7%, 28.2%)
	Saskatchewan	Stage 1	503	38	7.6%	(5.6%, 10.2%)
	Saskatchewan	Stage 2	111	43	38.7%	(30.2%, 48.0%)
	Saskatchewan	Stage 3	773	317	41.0%	(37.6%, 44.5%)
	Saskatchewan	Stage 4	1,704	546	32.0%	(29.9%, 34.3%)
	Saskatchewan	No recorded stage	148	20	13.5%	(8.9%, 20.0%)
	Manitoba	Stage 1	766	20	2.6%	(1.7%, 4.0%)
	Manitoba	Stage 2	318	100	31.4%	(26.6%, 36.7%)
	Manitoba	Stage 3	792	350	44.2%	(40.8%, 47.7%)
	Manitoba	Stage 4	1,998	560	28.0%	(26.1%, 30.0%)
	Manitoba	No recorded stage	46	<10		
	Prince Edward Island	Stage 1	109	0	0.0%	(0.0%, 3.4%)
	Prince Edward Island	Stage 2	37	16	43.2%	(28.7%, 59.1%)
	Prince Edward Island	Stage 3	143	75	52.4%	(44.3%, 60.5%)
	Prince Edward Island	Stage 4	321	95	29.6%	(24.9%, 34.8%)
	Prince Edward Island	No recorded stage	12	0	0.0%	(-0.0%, 24.2%)
	Nova Scotia	Stage 1	903	46	5.1%	(3.8%, 6.7%)
	Nova Scotia	Stage 2	366	107	29.2%	(24.8%, 34.1%)
	Nova Scotia	Stage 3	749	336	44.9%	(41.3%, 48.4%)
	Nova Scotia	Stage 4	2,236	615	27.5%	(25.7%, 29.4%)
	Nova Scotia	No recorded stage	86	0	0.0%	(0.0%, 4.3%)
Australia	New South Wales	Localised	3,209	673	21.0%	(19.6%, 22.4%)
	New South Wales	Regional	3,411	1,862	54.6%	(52.9%, 56.3%)
	New South Wales	Distant	7,435	3,619	48.7%	(47.5%, 49.8%)
	New South Wales	No recorded stage	2,279	801	35.1%	(33.2%, 37.1%)
	Victoria	No recorded stage	10,620	4,206	39.6%	(38.7%, 40.5%)

Appendix 2 Table 1b. Jurisdictional radiotherapy counts used to produce Figure 1 and Table 2. Counts between 1 and 9 are suppressed; where suppression has been applied, other counts are rounded to the nearest 10.

				Received treatment		
**Country**	**Jurisdiction**	**Stage at diagnosis**	**Patients**	**N**	**%**	**(95% CI)**
United Kingdom	England	Stage 1	19,368	4,690	24.2%	(23.6%, 24.8%)
	England	Stage 2	9,742	3,179	32.6%	(31.7%, 33.6%)
	England	Stage 3	26,334	12,994	49.3%	(48.7%, 49.9%)
	England	Stage 4	68,141	21,789	32.0%	(31.6%, 32.3%)
	England	No recorded stage	11,811	1,224	10.4%	(9.8%, 10.9%)
	Northern Ireland	Stage 1	675	152	22.5%	(19.5%, 25.8%)
	Northern Ireland	Stage 2	352	111	31.5%	(26.9%, 36.6%)
	Northern Ireland	Stage 3	1,053	594	56.4%	(53.4%, 59.4%)
	Northern Ireland	Stage 4	2,059	818	39.7%	(37.6%, 41.9%)
	Northern Ireland	No recorded stage	455	59	13.0%	(10.2%, 16.4%)
	Scotland	Stage 1	2,781	719	25.9%	(24.3%, 27.5%)
	Scotland	Stage 2	1,249	474	38.0%	(35.3%, 40.7%)
	Scotland	Stage 3	3,809	1,930	50.7%	(49.1%, 52.3%)
	Scotland	Stage 4	9,245	2,786	30.1%	(29.2%, 31.1%)
	Scotland	No recorded stage	1,578	247	15.7%	(13.9%, 17.5%)
	Wales	Stage 1	1,326	279	21.0%	(18.9%, 23.3%)
	Wales	Stage 2	714	252	35.3%	(31.9%, 38.9%)
	Wales	Stage 3	2,074	1,052	50.7%	(48.6%, 52.9%)
	Wales	Stage 4	4,400	1,479	33.6%	(32.2%, 35.0%)
	Wales	No recorded stage	851	152	17.9%	(15.4%, 20.6%)
Norway	Norway	Localised	1,979	674	34.1%	(32.0%, 36.2%)
	Norway	Regional	3,259	1,769	54.3%	(52.6%, 56.0%)
	Norway	Distant	4,895	2,297	46.9%	(45.5%, 48.3%)
	Norway	No recorded stage	1,414	616	43.6%	(41.0%, 46.2%)
Canada	Alberta	Stage 1	1,765	445	25.2%	(23.2%, 27.3%)
	Alberta	Stage 2	747	228	30.5%	(27.3%, 33.9%)
	Alberta	Stage 3	1,666	972	58.3%	(56.0%, 60.7%)
	Alberta	Stage 4	5,111	2,396	46.9%	(45.5%, 48.2%)
	Alberta	No recorded stage	119	17	14.3%	(9.1%, 21.7%)
	British Columbia	Stage 1	2,298	467	20.3%	(18.7%, 22.0%)
	British Columbia	Stage 2	1,083	349	32.2%	(29.5%, 35.1%)
	British Columbia	Stage 3	3,005	1,724	57.4%	(55.6%, 59.1%)
	British Columbia	Stage 4	7,561	3,696	48.9%	(47.8%, 50.0%)
	British Columbia	No recorded stage	792	45	5.7%	(4.3%, 7.5%)
	Ontario	Stage 1	7,035	2,158	30.7%	(29.6%, 31.8%)
	Ontario	Stage 2	2,780	975	35.1%	(33.3%, 36.9%)
	Ontario	Stage 3	6,801	4,490	66.0%	(64.9%, 67.1%)
	Ontario	Stage 4	17,017	6,015	35.3%	(34.6%, 36.1%)
	Ontario	No recorded stage	1,459	219	15.0%	(13.3%, 16.9%)
	Saskatchewan	Stage 1	503	62	12.3%	(9.7%, 15.5%)
	Saskatchewan	Stage 2	111	29	26.1%	(18.9%, 35.0%)
	Saskatchewan	Stage 3	773	323	41.8%	(38.4%, 45.3%)
	Saskatchewan	Stage 4	1,704	638	37.4%	(35.2%, 39.8%)
	Saskatchewan	No recorded stage	148	18	12.2%	(7.8%, 18.4%)
	Manitoba	Stage 1	766	131	17.1%	(14.6%, 19.9%)
	Manitoba	Stage 2	318	93	29.2%	(24.5%, 34.5%)
	Manitoba	Stage 3	792	458	57.8%	(54.4%, 61.2%)
	Manitoba	Stage 4	1,998	1,032	51.7%	(49.5%, 53.8%)
	Manitoba	No recorded stage	46	12	26.1%	(15.6%, 40.3%)
	Prince Edward Island	Stage 1	109	40	36.7%	(28.2%, 46.1%)
	Prince Edward Island	Stage 2	37	<10		
	Prince Edward Island	Stage 3	143	90	62.9%	(54.8%, 70.4%)
	Prince Edward Island	Stage 4	321	160	49.8%	(44.4%, 55.3%)
	Prince Edward Island	No recorded stage	12	0	0.0%	(0.0%, 24.2%)
	New Brunswick	Stage 1	892	140	15.7%	(13.5%, 18.2%)
	New Brunswick	Stage 2	334	100	29.9%	(25.3%, 35.1%)
	New Brunswick	Stage 3	776	420	54.1%	(50.6%, 57.6%)
	New Brunswick	Stage 4	1,659	650	39.2%	(36.9%, 41.6%)
	New Brunswick	No recorded stage	37	<10		
	Newfoundland & Labrador	Stage 1	495	199	40.2%	(36.0%, 44.6%)
	Newfoundland & Labrador	Stage 2	167	73	43.7%	(36.4%, 51.3%)
	Newfoundland & Labrador	Stage 3	444	328	73.9%	(69.6%, 77.7%)
	Newfoundland & Labrador	Stage 4	1,010	488	48.3%	(45.2%, 51.4%)
	Newfoundland & Labrador	No recorded stage	81	17	21.0%	(13.5%, 31.1%)
	Nova Scotia	Stage 1	903	257	28.5%	(25.6%, 31.5%)

	Nova Scotia	Stage 2	366	137	37.4%	(32.6%, 42.5%)
	Nova Scotia	Stage 3	749	469	62.6%	(59.1%, 66.0%)
	Nova Scotia	Stage 4	2,236	1,048	46.9%	(44.8%, 48.9%)
	Nova Scotia	No recorded stage	86	11	12.8%	(7.3%, 21.5%)
Australia	New South Wales	Localised	3,209	932	29.0%	(27.5%, 30.6%)
	New South Wales	Regional	3,411	1,789	52.4%	(50.8%, 54.1%)
	New South Wales	Distant	7,435	4,128	55.5%	(54.4%, 56.6%)
	New South Wales	No recorded stage	2,279	1,034	45.4%	(43.3%, 47.4%)
	Victoria	No recorded stage	10,620	4,332	40.8%	(39.9%, 41.7%)

Appendix 2 Table 2a. Chemotherapy all stages age-specific jurisdictional treatment counts used to produce Figure 2. Counts between 1 and 9 are suppressed; where suppression has been applied, other counts are rounded to the nearest 10. For SK-MB, suppression was applied to the count for Manitoba.

				Received treatment		
**Country**	**Jurisdiction**	**Age at diagnosis**	**Patients**	**N**	**%**	**(95% CI)**
Chemo - All stages						
United Kingdom	England	15-64	30,543	14,819	48.5%	(48.0%, 49.1%)
United Kingdom	England	65-74	45,107	16,231	36.0%	(35.5%, 36.4%)
United Kingdom	England	75-84	42,119	6,962	16.5%	(16.2%, 16.9%)
United Kingdom	England	85-99	17,627	398	2.3%	(2.0%, 2.5%)
United Kingdom	NI	15-64	1,466	620	42.3%	(39.8%, 44.8%)
United Kingdom	NI	65-74	2,022	540	26.7%	(24.8%, 28.7%)
United Kingdom	NI	75-84	1,724	150	8.7%	(7.5%, 10.1%)
United Kingdom	NI	85-99	540	<10		
United Kingdom	Scotland	15-64	4,319	2,004	46.4%	(44.9%, 47.9%)
United Kingdom	Scotland	65-74	6,461	1,829	28.3%	(27.2%, 29.4%)
United Kingdom	Scotland	75-84	5,818	634	10.9%	(10.1%, 11.7%)
United Kingdom	Scotland	85-99	2,064	29	1.4%	(1.0%, 2.0%)
United Kingdom	Wales	15-64	2,082	998	47.9%	(45.8%, 50.1%)
United Kingdom	Wales	65-74	3,335	1,207	36.2%	(34.6%, 37.8%)
United Kingdom	Wales	75-84	2,853	483	16.9%	(15.6%, 18.3%)
United Kingdom	Wales	85-99	1,095	19	1.7%	(1.1%, 2.7%)
Norway	Norway	15-64	2,989	1,900	63.6%	(61.8%, 65.3%)
Norway	Norway	65-74	4,589	2,395	52.2%	(50.7%, 53.6%)
Norway	Norway	75-84	3,055	881	28.8%	(27.3%, 30.5%)
Norway	Norway	85-99	914	52	5.7%	(4.4%, 7.4%)
Canada	Alberta	15-64	2,870	1,323	46.1%	(44.3%, 47.9%)
Canada	Alberta	65-74	3,187	1,017	31.9%	(30.3%, 33.6%)
Canada	Alberta	75-84	2,514	427	17.0%	(15.6%, 18.5%)
Canada	Alberta	85-99	837	37	4.4%	(3.2%, 6.0%)
Canada	BC	15-64	3,975	1,849	46.5%	(45.0%, 48.1%)
Canada	BC	65-74	4,744	1,561	32.9%	(31.6%, 34.3%)
Canada	BC	75-84	4,288	718	16.7%	(15.7%, 17.9%)
Canada	BC	85-99	1,732	66	3.8%	(3.0%, 4.8%)
Canada	Ontario	15-64	10,741	5,954	55.4%	(54.5%, 56.4%)
Canada	Ontario	65-74	12,271	5,608	45.7%	(44.8%, 46.6%)
Canada	Ontario	75-84	9,569	2,774	29.0%	(28.1%, 29.9%)
Canada	Ontario	85-99	2,511	370	14.7%	(13.4%, 16.2%)
Canada	SK-MB	15-64	1,933	860	44.5%	(42.3%, 46.7%)
Canada	SK-MB	65-74	2,475	821	33.2%	(31.3%, 35.1%)
Canada	SK-MB	75-84	2,009	302	15.0%	(13.5%, 16.7%)
Canada	SK-MB	85-99	742	<30		
Canada	Atl. Canada	15-64	1,376	582	42.3%	(39.7%, 44.9%)
Canada	Atl. Canada	65-74	1,793	560	31.2%	(29.1%, 33.4%)
Canada	Atl. Canada	75-84	1,345	155	11.5%	(9.9%, 13.3%)
Canada	Atl. Canada	85-99	448	0	0.0%	(0.0%, 0.9%)
Australia	NSW	15-64	4,769	2,901	60.8%	(59.4%, 62.2%)
Australia	NSW	65-74	5,528	2,713	49.1%	(47.8%, 50.4%)
Australia	NSW	75-84	4,536	1,217	26.8%	(25.6%, 28.1%)
Australia	NSW	85-99	1,501	124	8.3%	(7.0%, 9.8%)
Australia	Victoria	15-64	2,851	1,626	57.0%	(55.2%, 58.8%)
Australia	Victoria	65-74	3,527	1,619	45.9%	(44.3%, 47.6%)
Australia	Victoria	75-84	3,111	877	28.2%	(26.6%, 29.8%)
Australia	Victoria	85-99	1,131	84	7.4%	(6.0%, 9.1%)

Appendix 2 Table 2b. Chemotherapy stages 1-3 or L-R age-specific jurisdictional treatment counts used to produce Figure 2. Counts between 1 and 9 are suppressed; where suppression has been applied, other counts are rounded to the nearest 10.

				Received treatment		
Country	Jurisdiction	Age at diagnosis	Patients	N	%	(95% CI)
Chemo - Stages 1-3 or L-R						
United Kingdom	England	15-64	12,216	5,870	48.1%	(47.2%, 48.9%)
United Kingdom	England	65-74	19,250	6,918	35.9%	(35.3%, 36.6%)
United Kingdom	England	75-84	17,787	2,915	16.4%	(15.9%, 16.9%)
United Kingdom	England	85-99	6,191	145	2.3%	(2.0%, 2.7%)
United Kingdom	NI	15-64	668	270	40.4%	(36.8%, 44.2%)
United Kingdom	NI	65-74	937	254	27.1%	(24.4%, 30.0%)
United Kingdom	NI	75-84	789	66	8.4%	(6.6%, 10.5%)
United Kingdom	NI	85-99	223	0	0.0%	(0.0%, 1.7%)
United Kingdom	Scotland	15-64	1,821	780	42.8%	(40.6%, 45.1%)
United Kingdom	Scotland	65-74	2,751	700	25.4%	(23.9%, 27.1%)
United Kingdom	Scotland	75-84	2,514	240	9.5%	(8.5%, 10.8%)
United Kingdom	Scotland	85-99	753	<10		
United Kingdom	Wales	15-64	835	420	50.3%	(46.9%, 53.7%)
United Kingdom	Wales	65-74	1,560	600	38.5%	(36.1%, 40.9%)
United Kingdom	Wales	75-84	1,280	230	18.0%	(16.0%, 20.2%)
United Kingdom	Wales	85-99	439	<10		
Norway	Norway	15-64	1,427	809	56.7%	(54.1%, 59.2%)
Norway	Norway	65-74	2,214	1,005	45.4%	(43.3%, 47.5%)
Norway	Norway	75-84	1,311	330	25.2%	(22.9%, 27.6%)
Norway	Norway	85-99	286	23	8.0%	(5.4%, 11.8%)
Canada	Alberta	15-64	1,193	490	41.1%	(38.3%, 43.9%)
Canada	Alberta	65-74	1,491	420	28.2%	(25.9%, 30.5%)
Canada	Alberta	75-84	1,167	160	13.7%	(11.9%, 15.8%)
Canada	Alberta	85-99	327	<10		
Canada	BC	15-64	1,668	699	41.9%	(39.6%, 44.3%)
Canada	BC	65-74	2,147	634	29.5%	(27.6%, 31.5%)
Canada	BC	75-84	1,949	255	13.1%	(11.7%, 14.7%)
Canada	BC	85-99	622	16	2.6%	(1.6%, 4.1%)
Canada	Ontario	15-64	4,754	2,379	50.0%	(48.6%, 51.5%)
Canada	Ontario	65-74	6,061	2,509	41.4%	(40.2%, 42.6%)
Canada	Ontario	75-84	4,707	1,200	25.5%	(24.3%, 26.8%)
Canada	Ontario	85-99	1,094	134	12.2%	(10.4%, 14.3%)
Canada	SK-MB	15-64	774	339	43.8%	(40.3%, 47.3%)
Canada	SK-MB	65-74	1,127	372	33.0%	(30.3%, 35.8%)
Canada	SK-MB	75-84	1,004	149	14.8%	(12.8%, 17.2%)
Canada	SK-MB	85-99	358	0	0.0%	(0.0%, 1.1%)
Canada	Atl. Canada	15-64	594	225	37.9%	(34.1%, 41.8%)
Canada	Atl. Canada	65-74	899	286	31.8%	(28.9%, 34.9%)
Canada	Atl. Canada	75-84	634	73	11.5%	(9.3%, 14.2%)
Canada	Atl. Canada	85-99	180	0	0.0%	(0.0%, 2.1%)
Australia	NSW	15-64	1,875	992	52.9%	(50.6%, 55.2%)
Australia	NSW	65-74	2,442	1,078	44.1%	(42.2%, 46.1%)
Australia	NSW	75-84	1,814	426	23.5%	(21.6%, 25.5%)
Australia	NSW	85-99	489	39	8.0%	(5.9%, 10.7%)

Appendix 2 Table 2c. Chemotherapy stage 4 or distant age-specific jurisdictional treatment counts used to produce Figure 2. Counts between 1 and 9 are suppressed; where suppression has been applied, other counts are rounded to the nearest 10. For SK-MB, suppression was applied to the count for Saskatchewan.

				Received treatment		
**Country**	**Jurisdiction**	**Age at diagnosis**	**Patients**	**N**	**%**	**(95% CI)**
Chemo - Stage 4 or distant						
United Kingdom	England	15-64	16,563	8,578	51.8%	(51.0%, 52.6%)
United Kingdom	England	65-74	23,004	8,957	38.9%	(38.3%, 39.6%)
United Kingdom	England	75-84	20,279	3,889	19.2%	(18.6%, 19.7%)
United Kingdom	England	85-99	8,295	243	2.9%	(2.6%, 3.3%)
United Kingdom	NI	15-64	720	340	47.2%	(43.6%, 50.9%)
United Kingdom	NI	65-74	961	270	28.1%	(25.3%, 31.0%)
United Kingdom	NI	75-84	717	70	9.8%	(7.8%, 12.2%)
United Kingdom	NI	85-99	196	<10		
United Kingdom	Scotland	15-64	2,330	1,188	51.0%	(49.0%, 53.0%)
United Kingdom	Scotland	65-74	3,338	1,102	33.0%	(31.4%, 34.6%)
United Kingdom	Scotland	75-84	2,703	382	14.1%	(12.9%, 15.5%)
United Kingdom	Scotland	85-99	874	21	2.4%	(1.6%, 3.6%)
United Kingdom	Wales	15-64	1,092	535	49.0%	(46.0%, 52.0%)
United Kingdom	Wales	65-74	1,552	559	36.0%	(33.7%, 38.4%)
United Kingdom	Wales	75-84	1,287	223	17.3%	(15.4%, 19.5%)
United Kingdom	Wales	85-99	469	11	2.3%	(1.3%, 4.2%)
Norway	Norway	15-64	1,327	953	71.8%	(69.3%, 74.2%)
Norway	Norway	65-74	1,940	1,197	61.7%	(59.5%, 63.8%)
Norway	Norway	75-84	1,252	458	36.6%	(34.0%, 39.3%)
Norway	Norway	85-99	376	25	6.6%	(4.5%, 9.6%)
Canada	Alberta	15-64	1,656	827	49.9%	(47.5%, 52.3%)
Canada	Alberta	65-74	1,669	594	35.6%	(33.3%, 37.9%)
Canada	Alberta	75-84	1,300	261	20.1%	(18.0%, 22.3%)
Canada	Alberta	85-99	486	32	6.6%	(4.7%, 9.1%)
Canada	BC	15-64	2,178	1,128	51.8%	(49.7%, 53.9%)
Canada	BC	65-74	2,416	913	37.8%	(35.9%, 39.7%)
Canada	BC	75-84	2,057	448	21.8%	(20.0%, 23.6%)
Canada	BC	85-99	910	48	5.3%	(4.0%, 6.9%)
Canada	Ontario	15-64	5,582	3,428	61.4%	(60.1%, 62.7%)
Canada	Ontario	65-74	5,745	2,965	51.6%	(50.3%, 52.9%)
Canada	Ontario	75-84	4,435	1,493	33.7%	(32.3%, 35.1%)
Canada	Ontario	85-99	1,255	220	17.5%	(15.5%, 19.7%)
Canada	SK-MB	15-64	1,112	517	46.5%	(43.6%, 49.4%)
Canada	SK-MB	65-74	1,303	445	34.2%	(31.6%, 36.8%)
Canada	SK-MB	75-84	936	139	14.9%	(12.7%, 17.3%)
Canada	SK-MB	85-99	351	<30		
Canada	Atl. Canada	15-64	769	356	46.3%	(42.8%, 49.8%)
Canada	Atl. Canada	65-74	872	271	31.1%	(28.1%, 34.2%)
Canada	Atl. Canada	75-84	685	81	11.8%	(9.6%, 14.5%)
Canada	Atl. Canada	85-99	231	0	0.0%	(-0.0%, 1.6%)
Australia	NSW	15-64	2,395	1,608	67.1%	(65.2%, 69.0%)
Australia	NSW	65-74	2,445	1,339	54.8%	(52.8%, 56.7%)
Australia	NSW	75-84	1,967	620	31.5%	(29.5%, 33.6%)
Australia	NSW	85-99	628	52	8.3%	(6.4%, 10.7%)

Appendix 2 Table 2d. Radiotherapy all stages age-specific jurisdictional treatment counts used to produce Figure 2.

				Received treatment		
Country	Jurisdiction	Age at diagnosis	Patients	N	%	(95% CI)
Radio - All stages						
United Kingdom	England	15-64	30,543	13,277	43.5%	(42.9%, 44.0%)
United Kingdom	England	65-74	45,107	16,384	36.3%	(35.9%, 36.8%)
United Kingdom	England	75-84	42,119	11,674	27.7%	(27.3%, 28.1%)
United Kingdom	England	85-99	17,627	2,541	14.4%	(13.9%, 14.9%)
United Kingdom	NI	15-64	1,180	619	52.5%	(49.6%, 55.3%)
United Kingdom	NI	65-74	1,621	655	40.4%	(38.0%, 42.8%)
United Kingdom	NI	75-84	1,364	413	30.3%	(27.9%, 32.8%)
United Kingdom	NI	85-99	429	47	11.0%	(8.3%, 14.3%)
United Kingdom	Scotland	15-64	4,319	1,905	44.1%	(42.6%, 45.6%)
United Kingdom	Scotland	65-74	6,461	2,388	37.0%	(35.8%, 38.1%)
United Kingdom	Scotland	75-84	5,818	1,610	27.7%	(26.5%, 28.8%)
United Kingdom	Scotland	85-99	2,064	253	12.3%	(10.9%, 13.7%)
United Kingdom	Wales	15-64	2,082	1,009	48.5%	(46.3%, 50.6%)
United Kingdom	Wales	65-74	3,335	1,295	38.8%	(37.2%, 40.5%)
United Kingdom	Wales	75-84	2,853	786	27.5%	(25.9%, 29.2%)
United Kingdom	Wales	85-99	1,095	124	11.3%	(9.6%, 13.3%)
Norway	Norway	15-64	2,989	1,669	55.8%	(54.1%, 57.6%)
Norway	Norway	65-74	4,589	2,190	47.7%	(46.3%, 49.2%)
Norway	Norway	75-84	3,055	1,271	41.6%	(39.9%, 43.4%)
Norway	Norway	85-99	914	226	24.7%	(22.0%, 27.6%)
Canada	Alberta	15-64	2,870	1,459	50.8%	(49.0%, 52.7%)
Canada	Alberta	65-74	3,187	1,426	44.7%	(43.0%, 46.5%)
Canada	Alberta	75-84	2,514	966	38.4%	(36.5%, 40.3%)
Canada	Alberta	85-99	837	207	24.7%	(21.9%, 27.8%)
Canada	BC	15-64	3,975	2,051	51.6%	(50.0%, 53.1%)
Canada	BC	65-74	4,744	2,165	45.6%	(44.2%, 47.1%)
Canada	BC	75-84	4,288	1,661	38.7%	(37.3%, 40.2%)
Canada	BC	85-99	1,732	404	23.3%	(21.4%, 25.4%)
Canada	Ontario	15-64	10,741	4,317	40.2%	(39.3%, 41.1%)
Canada	Ontario	65-74	12,271	4,851	39.5%	(38.7%, 40.4%)
Canada	Ontario	75-84	9,569	3,723	38.9%	(37.9%, 39.9%)
Canada	Ontario	85-99	2,511	966	38.5%	(36.6%, 40.4%)
Canada	SK-MB	15-64	1,933	974	50.4%	(48.2%, 52.6%)
Canada	SK-MB	65-74	2,475	1,052	42.5%	(40.6%, 44.5%)
Canada	SK-MB	75-84	2,009	638	31.8%	(29.8%, 33.8%)
Canada	SK-MB	85-99	742	132	17.8%	(15.2%, 20.7%)
Canada	Atl. Canada	15-64	3,113	1,566	50.3%	(48.5%, 52.1%)
Canada	Atl. Canada	65-74	3,934	1,726	43.9%	(42.3%, 45.4%)
Canada	Atl. Canada	75-84	2,885	1,156	40.1%	(38.3%, 41.9%)
Canada	Atl. Canada	85-99	925	189	20.4%	(18.0%, 23.2%)
Australia	NSW	15-64	4,769	2,742	57.5%	(56.1%, 58.9%)
Australia	NSW	65-74	5,528	2,733	49.4%	(48.1%, 50.8%)
Australia	NSW	75-84	4,536	1,945	42.9%	(41.4%, 44.3%)
Australia	NSW	85-99	1,501	463	30.8%	(28.6%, 33.2%)
Australia	Victoria	15-64	2,851	1,403	49.2%	(47.4%, 51.0%)
Australia	Victoria	65-74	3,527	1,485	42.1%	(40.5%, 43.7%)
Australia	Victoria	75-84	3,111	1,153	37.1%	(35.4%, 38.8%)
Australia	Victoria	85-99	1,131	291	25.7%	(23.3%, 28.4%)

Appendix 2 Table 2e. Radiotherapy stages 1-3 or L-R age-specific jurisdictional treatment counts used to produce Figure 2.

				Received treatment		
**Country**	**Jurisdiction**	**Age at diagnosis**	**Patients**	**N**	**%**	**(95% CI)**
Radio - Stages 1-3 or L-R						
United Kingdom	England	15-64	12,216	5,284	43.3%	(42.4%, 44.1%)
United Kingdom	England	65-74	19,250	7,662	39.8%	(39.1%, 40.5%)
United Kingdom	England	75-84	17,787	6,387	35.9%	(35.2%, 36.6%)
United Kingdom	England	85-99	6,191	1,530	24.7%	(23.7%, 25.8%)
United Kingdom	NI	15-64	536	250	46.6%	(42.5%, 50.9%)
United Kingdom	NI	65-74	754	337	44.7%	(41.2%, 48.3%)
United Kingdom	NI	75-84	622	245	39.4%	(35.6%, 43.3%)
United Kingdom	NI	85-99	168	25	14.9%	(10.3%, 21.0%)
United Kingdom	Scotland	15-64	1,821	834	45.8%	(43.5%, 48.1%)
United Kingdom	Scotland	65-74	2,751	1,189	43.2%	(41.4%, 45.1%)
United Kingdom	Scotland	75-84	2,514	942	37.5%	(35.6%, 39.4%)
United Kingdom	Scotland	85-99	753	158	21.0%	(18.2%, 24.0%)
United Kingdom	Wales	15-64	835	414	49.6%	(46.2%, 53.0%)
United Kingdom	Wales	65-74	1,560	650	41.7%	(39.2%, 44.1%)
United Kingdom	Wales	75-84	1,280	452	35.3%	(32.7%, 38.0%)
United Kingdom	Wales	85-99	439	67	15.3%	(12.2%, 18.9%)
Norway	Norway	15-64	1,427	689	48.3%	(45.7%, 50.9%)
Norway	Norway	65-74	2,214	997	45.0%	(43.0%, 47.1%)
Norway	Norway	75-84	1,311	645	49.2%	(46.5%, 51.9%)
Norway	Norway	85-99	286	112	39.2%	(33.7%, 44.9%)
Canada	Alberta	15-64	1,193	459	38.5%	(35.8%, 41.3%)
Canada	Alberta	65-74	1,491	588	39.4%	(37.0%, 41.9%)
Canada	Alberta	75-84	1,167	482	41.3%	(38.5%, 44.2%)
Canada	Alberta	85-99	327	116	35.5%	(30.5%, 40.8%)
Canada	BC	15-64	1,668	692	41.5%	(39.1%, 43.9%)
Canada	BC	65-74	2,147	846	39.4%	(37.4%, 41.5%)
Canada	BC	75-84	1,949	793	40.7%	(38.5%, 42.9%)
Canada	BC	85-99	622	209	33.6%	(30.0%, 37.4%)
Canada	Ontario	15-64	4,754	2,011	42.3%	(40.9%, 43.7%)
Canada	Ontario	65-74	6,061	2,674	44.1%	(42.9%, 45.4%)
Canada	Ontario	75-84	4,707	2,299	48.8%	(47.4%, 50.3%)
Canada	Ontario	85-99	1,094	639	58.4%	(55.5%, 61.3%)
Canada	SK-MB	15-64	774	304	39.3%	(35.9%, 42.8%)
Canada	SK-MB	65-74	1,127	404	35.8%	(33.1%, 38.7%)
Canada	SK-MB	75-84	1,004	308	30.7%	(27.9%, 33.6%)
Canada	SK-MB	85-99	358	80	22.3%	(18.3%, 26.9%)
Canada	Atl. Canada	15-64	1,465	626	42.7%	(40.2%, 45.3%)
Canada	Atl. Canada	65-74	2,053	851	41.5%	(39.3%, 43.6%)
Canada	Atl. Canada	75-84	1,487	669	45.0%	(42.5%, 47.5%)
Canada	Atl. Canada	85-99	410	110	26.8%	(22.8%, 31.3%)
Australia	NSW	15-64	1,875	814	43.4%	(41.2%, 45.7%)
Australia	NSW	65-74	2,442	984	40.3%	(38.4%, 42.3%)
Australia	NSW	75-84	1,814	742	40.9%	(38.7%, 43.2%)
Australia	NSW	85-99	489	181	37.0%	(32.9%, 41.4%)

Appendix 2 Table 2f. Radiotherapy stage 4 or distant age-specific jurisdictional treatment counts used to produce Figure 2.

				Received treatment		
Country	Jurisdiction	Age at diagnosis	Patients	N	%	(95% CI)
Radio - Stage 4 or distant						
United Kingdom	England	15-64	16,563	7,634	46.1%	(45.3%, 46.9%)
United Kingdom	England	65-74	23,004	8,297	36.1%	(35.4%, 36.7%)
United Kingdom	England	75-84	20,279	4,928	24.3%	(23.7%, 24.9%)
United Kingdom	England	85-99	8,295	930	11.2%	(10.6%, 11.9%)
United Kingdom	NI	15-64	576	349	60.6%	(56.5%, 64.5%)
United Kingdom	NI	65-74	762	302	39.6%	(36.2%, 43.1%)
United Kingdom	NI	75-84	563	149	26.5%	(23.0%, 30.3%)
United Kingdom	NI	85-99	158	18	11.4%	(7.3%, 17.3%)
United Kingdom	Scotland	15-64	2,330	1,019	43.7%	(41.7%, 45.8%)
United Kingdom	Scotland	65-74	3,338	1,096	32.8%	(31.3%, 34.4%)
United Kingdom	Scotland	75-84	2,703	591	21.9%	(20.3%, 23.5%)
United Kingdom	Scotland	85-99	874	80	9.2%	(7.4%, 11.2%)
United Kingdom	Wales	15-64	1,092	544	49.8%	(46.9%, 52.8%)
United Kingdom	Wales	65-74	1,552	591	38.1%	(35.7%, 40.5%)
United Kingdom	Wales	75-84	1,287	296	23.0%	(20.8%, 25.4%)
United Kingdom	Wales	85-99	469	48	10.2%	(7.8%, 13.3%)
Norway	Norway	15-64	1,327	837	63.1%	(60.4%, 65.6%)
Norway	Norway	65-74	1,940	963	49.6%	(47.4%, 51.9%)
Norway	Norway	75-84	1,252	420	33.5%	(31.0%, 36.2%)
Norway	Norway	85-99	376	77	20.5%	(16.7%, 24.8%)
Canada	Alberta	15-64	1,656	998	60.3%	(57.9%, 62.6%)
Canada	Alberta	65-74	1,669	833	49.9%	(47.5%, 52.3%)
Canada	Alberta	75-84	1,300	476	36.6%	(34.0%, 39.3%)
Canada	Alberta	85-99	486	89	18.3%	(15.1%, 22.0%)
Canada	BC	15-64	2,178	1,347	61.8%	(59.8%, 63.9%)
Canada	BC	65-74	2,416	1,309	54.2%	(52.2%, 56.2%)
Canada	BC	75-84	2,057	849	41.3%	(39.2%, 43.4%)
Canada	BC	85-99	910	191	21.0%	(18.5%, 23.8%)
Canada	Ontario	15-64	5,582	2,235	40.0%	(38.8%, 41.3%)
Canada	Ontario	65-74	5,745	2,100	36.6%	(35.3%, 37.8%)
Canada	Ontario	75-84	4,435	1,362	30.7%	(29.4%, 32.1%)
Canada	Ontario	85-99	1,255	318	25.3%	(23.0%, 27.8%)
Canada	SK-MB	15-64	1,112	661	59.4%	(56.5%, 62.3%)
Canada	SK-MB	65-74	1,303	638	49.0%	(46.3%, 51.7%)
Canada	SK-MB	75-84	936	320	34.2%	(31.2%, 37.3%)
Canada	SK-MB	85-99	351	51	14.5%	(11.2%, 18.6%)
Canada	Atl. Canada	15-64	1,614	932	57.7%	(55.3%, 60.1%)
Canada	Atl. Canada	65-74	1,828	859	47.0%	(44.7%, 49.3%)
Canada	Atl. Canada	75-84	1,336	478	35.8%	(33.3%, 38.4%)
Canada	Atl. Canada	85-99	448	66	14.7%	(11.8%, 18.3%)
Australia	NSW	15-64	2,395	1,661	69.4%	(67.5%, 71.2%)
Australia	NSW	65-74	2,445	1,422	58.2%	(56.2%, 60.1%)
Australia	NSW	75-84	1,967	878	44.6%	(42.5%, 46.8%)
Australia	NSW	85-99	628	167	26.6%	(23.3%, 30.2%)

Appendix 2 Table 3a. Sex-specific jurisdictional chemotherapy counts used to produce Figure 3.

				Received treatment		
Country	Jurisdiction	Sex	Patients	N	%	(95% CI)
**Chemotherapy - All stages**						
United Kingdom	England	F	63,577	17,871	28.1%	(27.8%, 28.5%)
United Kingdom	England	M	71,819	20,539	28.6%	(28.3%, 28.9%)
United Kingdom	Northern Ireland	F	2,731	610	22.3%	(20.8%, 23.9%)
United Kingdom	Northern Ireland	M	3,021	706	23.4%	(21.9%, 24.9%)
United Kingdom	Scotland	F	9,473	2,317	24.5%	(23.6%, 25.3%)
United Kingdom	Scotland	M	9,189	2,179	23.7%	(22.9%, 24.6%)
United Kingdom	Wales	F	4,390	1,272	29.0%	(27.7%, 30.3%)
United Kingdom	Wales	M	4,975	1,435	28.8%	(27.6%, 30.1%)
Norway	Norway	F	5,459	2,435	44.6%	(43.3%, 45.9%)
Norway	Norway	M	6,088	2,793	45.9%	(44.6%, 47.1%)
Canada	Alberta	F	4,729	1,435	30.3%	(29.1%, 31.7%)
Canada	Alberta	M	4,679	1,369	29.3%	(28.0%, 30.6%)
Canada	British Columbia	F	7,532	2,247	29.8%	(28.8%, 30.9%)
Canada	British Columbia	M	7,207	1,947	27.0%	(26.0%, 28.1%)
Canada	Ontario	F	17,399	7,304	42.0%	(41.2%, 42.7%)
Canada	Ontario	M	17,693	7,402	41.8%	(41.1%, 42.6%)
Canada	Saskatchewan-Manitoba	F	3,676	1,017	27.7%	(26.2%, 29.1%)
Canada	Saskatchewan-Manitoba	M	3,483	990	28.4%	(27.0%, 29.9%)
Canada	Atlantic Canada	F	2,467	636	25.8%	(24.1%, 27.5%)
Canada	Atlantic Canada	M	2,495	664	26.6%	(24.9%, 28.4%)
Australia	New South Wales	F	7,020	3,022	43.0%	(41.9%, 44.2%)
Australia	New South Wales	M	9,314	3,933	42.2%	(41.2%, 43.2%)
Australia	Victoria	F	4,653	1,801	38.7%	(37.3%, 40.1%)
Australia	Victoria	M	5,967	2,405	40.3%	(39.1%, 41.6%)
**Chemotherapy - Stages 1-3 or L-R**						
United Kingdom	England	F	26,763	7,394	27.6%	(27.1%, 28.2%)
United Kingdom	England	M	28,681	8,454	29.5%	(29.0%, 30.0%)
United Kingdom	Northern Ireland	F	1,276	271	21.2%	(19.1%, 23.6%)
United Kingdom	Northern Ireland	M	1,341	319	23.8%	(21.6%, 26.1%)
United Kingdom	Scotland	F	4,116	887	21.6%	(20.3%, 22.8%)
United Kingdom	Scotland	M	3,723	826	22.2%	(20.9%, 23.5%)
United Kingdom	Wales	F	1,978	593	30.0%	(28.0%, 32.0%)
United Kingdom	Wales	M	2,136	656	30.7%	(28.8%, 32.7%)
Norway	Norway	F	2,490	1,001	40.2%	(38.3%, 42.1%)
Norway	Norway	M	2,748	1,166	42.4%	(40.6%, 44.3%)
Canada	Alberta	F	2,255	568	25.2%	(23.4%, 27.0%)
Canada	Alberta	M	1,923	511	26.6%	(24.6%, 28.6%)
Canada	British Columbia	F	3,342	850	25.4%	(24.0%, 26.9%)
Canada	British Columbia	M	3,044	754	24.8%	(23.3%, 26.3%)
Canada	Ontario	F	8,627	3,141	36.4%	(35.4%, 37.4%)
Canada	Ontario	M	7,989	3,081	38.6%	(37.5%, 39.6%)
Canada	Saskatchewan-Manitoba	F	1,745	452	25.9%	(23.9%, 28.0%)
Canada	Saskatchewan-Manitoba	M	1,518	418	27.5%	(25.3%, 29.8%)
Canada	Atlantic Canada	F	1,169	283	24.2%	(21.8%, 26.7%)
Canada	Atlantic Canada	M	1,138	302	26.5%	(24.1%, 29.2%)
Australia	New South Wales	F	3,028	1,136	37.5%	(35.8%, 39.3%)
Australia	New South Wales	M	3,592	1,399	38.9%	(37.4%, 40.6%)
**Chemotherapy - Stage 4 or distant**						
United Kingdom	England	F	31,191	10,041	32.2%	(31.7%, 32.7%)
United Kingdom	England	M	36,950	11,626	31.5%	(31.0%, 31.9%)
United Kingdom	Northern Ireland	F	1,182	322	27.2%	(24.8%, 29.9%)
United Kingdom	Northern Ireland	M	1,412	363	25.7%	(23.5%, 28.1%)
United Kingdom	Scotland	F	4,518	1,380	30.5%	(29.2%, 31.9%)
United Kingdom	Scotland	M	4,727	1,313	27.8%	(26.5%, 29.1%)
United Kingdom	Wales	F	1,998	627	31.4%	(29.4%, 33.5%)
United Kingdom	Wales	M	2,402	701	29.2%	(27.4%, 31.0%)
Norway	Norway	F	2,300	1,234	53.7%	(51.6%, 55.7%)
Norway	Norway	M	2,595	1,399	53.9%	(52.0%, 55.8%)
Canada	Alberta	F	2,429	864	35.6%	(33.7%, 37.5%)
Canada	Alberta	M	2,682	850	31.7%	(30.0%, 33.5%)
Canada	British Columbia	F	3,794	1,372	36.2%	(34.6%, 37.7%)
Canada	British Columbia	M	3,767	1,165	30.9%	(29.5%, 32.4%)
Canada	Ontario	F	8,031	3,954	49.2%	(48.1%, 50.3%)
Canada	Ontario	M	8,986	4,152	46.2%	(45.2%, 47.2%)
Canada	Saskatchewan-Manitoba	F	1,830	555	30.3%	(28.3%, 32.5%)

Canada	Saskatchewan-Manitoba	M	1,872	556	29.7%	(27.7%, 31.8%)
Canada	Atlantic Canada	F	1,245	350	28.1%	(25.7%, 30.7%)
Canada	Atlantic Canada	M	1,312	360	27.4%	(25.1%, 29.9%)
Australia	New South Wales	F	3,020	1,535	50.8%	(49.0%, 52.6%)
Australia	New South Wales	M	4,415	2,084	47.2%	(45.7%, 48.7%)

Appendix 2 Table 3b. Sex-specific jurisdictional radiotherapy counts used to produce Figure 3.

				Received treatment		
**Country**	**Jurisdiction**	**Sex**	**Patients**	**N**	**%**	**(95% CI)**
**Radiotherapy - All stages**						
United Kingdom	England	F	63,577	19,679	31.0%	(30.6%, 31.3%)
United Kingdom	England	M	71,819	24,197	33.7%	(33.3%, 34.0%)
United Kingdom	Northern Ireland	F	2,193	782	35.7%	(33.7%, 37.7%)
United Kingdom	Northern Ireland	M	2,401	952	39.7%	(37.7%, 41.6%)
United Kingdom	Scotland	F	9,473	3,043	32.1%	(31.2%, 33.1%)
United Kingdom	Scotland	M	9,189	3,113	33.9%	(32.9%, 34.9%)
United Kingdom	Wales	F	4,390	1,446	32.9%	(31.6%, 34.3%)
United Kingdom	Wales	M	4,975	1,768	35.5%	(34.2%, 36.9%)
Norway	Norway	F	5,459	2,460	45.1%	(43.7%, 46.4%)
Norway	Norway	M	6,088	2,896	47.6%	(46.3%, 48.8%)
Canada	Alberta	F	4,729	1,942	41.1%	(39.7%, 42.5%)
Canada	Alberta	M	4,679	2,116	45.2%	(43.8%, 46.7%)
Canada	British Columbia	F	7,532	3,170	42.1%	(41.0%, 43.2%)
Canada	British Columbia	M	7,207	3,111	43.2%	(42.0%, 44.3%)
Canada	Ontario	F	17,399	6,648	38.2%	(37.5%, 38.9%)
Canada	Ontario	M	17,693	7,209	40.7%	(40.0%, 41.5%)
Canada	Saskatchewan-Manitoba	F	3,676	1,419	38.6%	(37.0%, 40.2%)
Canada	Saskatchewan-Manitoba	M	3,483	1,377	39.5%	(37.9%, 41.2%)
Canada	Atlantic Canada	F	5,125	2,068	40.4%	(39.0%, 41.7%)
Canada	Atlantic Canada	M	5,732	2,569	44.8%	(43.5%, 46.1%)
Australia	New South Wales	F	7,020	3,207	45.7%	(44.5%, 46.9%)
Australia	New South Wales	M	9,314	4,676	50.2%	(49.2%, 51.2%)
Australia	Victoria	F	4,653	1,829	39.3%	(37.9%, 40.7%)
Australia	Victoria	M	5,967	2,503	41.9%	(40.7%, 43.2%)
**Radiotherapy - Stages 1-3 or L-R**						
United Kingdom	England	F	26,763	9,440	35.3%	(34.7%, 35.8%)
United Kingdom	England	M	28,681	11,423	39.8%	(39.3%, 40.4%)
United Kingdom	Northern Ireland	F	1,007	399	39.6%	(36.6%, 42.7%)
United Kingdom	Northern Ireland	M	1,073	458	42.7%	(39.8%, 45.7%)
United Kingdom	Scotland	F	4,116	1,590	38.6%	(37.2%, 40.1%)
United Kingdom	Scotland	M	3,723	1,533	41.2%	(39.6%, 42.8%)
United Kingdom	Wales	F	1,978	712	36.0%	(33.9%, 38.1%)
United Kingdom	Wales	M	2,136	871	40.8%	(38.7%, 42.9%)
Norway	Norway	F	2,490	1,113	44.7%	(42.8%, 46.7%)
Norway	Norway	M	2,748	1,330	48.4%	(46.5%, 50.3%)
Canada	Alberta	F	2,255	794	35.2%	(33.3%, 37.2%)
Canada	Alberta	M	1,923	851	44.3%	(42.0%, 46.5%)
Canada	British Columbia	F	3,342	1,292	38.7%	(37.0%, 40.3%)
Canada	British Columbia	M	3,044	1,248	41.0%	(39.3%, 42.8%)
Canada	Ontario	F	8,627	3,746	43.4%	(42.4%, 44.5%)
Canada	Ontario	M	7,989	3,877	48.5%	(47.4%, 49.6%)
Canada	Saskatchewan-Manitoba	F	1,745	580	33.2%	(31.1%, 35.5%)
Canada	Saskatchewan-Manitoba	M	1,518	516	34.0%	(31.7%, 36.4%)
Canada	Atlantic Canada	F	2,667	994	37.3%	(35.5%, 39.1%)
Canada	Atlantic Canada	M	2,748	1,267	46.1%	(44.2%, 48.0%)
Australia	New South Wales	F	3,028	1,127	37.2%	(35.5%, 39.0%)
Australia	New South Wales	M	3,592	1,594	44.4%	(42.8%, 46.0%)
**Radiotherapy - Stage 4 or distant**						
United Kingdom	England	F	31,191	9,689	31.1%	(30.6%, 31.6%)
United Kingdom	England	M	36,950	12,100	32.7%	(32.3%, 33.2%)
United Kingdom	Northern Ireland	F	950	358	37.7%	(34.7%, 40.8%)
United Kingdom	Northern Ireland	M	1,109	460	41.5%	(38.6%, 44.4%)
United Kingdom	Scotland	F	4,518	1,329	29.4%	(28.1%, 30.8%)
United Kingdom	Scotland	M	4,727	1,457	30.8%	(29.5%, 32.2%)
United Kingdom	Wales	F	1,998	664	33.2%	(31.2%, 35.3%)
United Kingdom	Wales	M	2,402	815	33.9%	(32.1%, 35.8%)
Norway	Norway	F	2,300	1,064	46.3%	(44.2%, 48.3%)
Norway	Norway	M	2,595	1,233	47.5%	(45.6%, 49.4%)
Canada	Alberta	F	2,429	1,145	47.1%	(45.2%, 49.1%)
Canada	Alberta	M	2,682	1,251	46.6%	(44.8%, 48.5%)
Canada	British Columbia	F	3,794	1,851	48.8%	(47.2%, 50.4%)
Canada	British Columbia	M	3,767	1,845	49.0%	(47.4%, 50.6%)
Canada	Ontario	F	8,031	2,794	34.8%	(33.8%, 35.8%)
Canada	Ontario	M	8,986	3,221	35.8%	(34.9%, 36.8%)
Canada	Saskatchewan-Manitoba	F	1,830	824	45.0%	(42.8%, 47.3%)
Canada	Saskatchewan-Manitoba	M	1,872	846	45.2%	(42.9%, 47.5%)
Canada	Atlantic Canada	F	2,352	1,056	44.9%	(42.9%, 46.9%)
Canada	Atlantic Canada	M	2,874	1,284	44.7%	(42.9%, 46.5%)
Australia	New South Wales	F	3,020	1,667	55.2%	(53.4%, 57.0%)

Australia	New South Wales	M	4,415	2,461	55.7%	(54.3%, 57.2%)

Appendix 2 Table 4. Key centiles of time-to-treatment aggregated to country-level, data relate to Figure 4.

		All lung cancers		Stages 1-3 or L-R		Stage 4 or distant	
Country	Percentile of those treated	Days	% treated	Days	% treated	Days	% treated
		Lung - Chemotherapy					
United Kingdom	25	23	6.6%	27	6.4%	21	7.3%
United Kingdom	50	37	14.3%	45	14.3%	33	16.1%
United Kingdom	75	61	20.3%	83	20.3%	50	22.8%
United Kingdom	90	122	25.0%	147	24.9%	98	28.1%
Norway	25	13	11.3%	19	10.3%	12	13.4%
Norway	50	27	22.6%	39	20.7%	22	26.9%
Norway	75	56	34.0%	76	31.0%	41	40.3%
Norway	90	97	40.7%	112	37.2%	73	48.4%
Canada	25	28	8.6%	35	7.8%	25	9.6%
Canada	50	52	17.4%	62	15.7%	45	19.5%
Canada	75	92	26.2%	108	23.6%	76	29.3%
Canada	90	163	31.6%	164	28.5%	131	35.5%
Australia	25	21	10.4%	25	9.6%	18	12.2%
Australia	50	39	20.7%	44	19.1%	34	24.3%
Australia	75	64	31.1%	75	28.7%	58	36.5%
Australia	90	108	37.3%	119	34.5%	94	43.8%
		Lung - Radiotherapy					
United Kingdom	25	34	7.9%	47	8.8%	22	7.9%
United Kingdom	50	72	16.3%	90	18.9%	49	16.0%
United Kingdom	75	142	24.5%	148	28.5%	133	24.1%
United Kingdom	90	200	29.5%	207	34.2%	192	28.9%
Norway	25	28	11.6%	34	11.7%	22	11.7%
Norway	50	47	23.2%	49	23.3%	44	23.5%
Norway	75	105	34.8%	94	35.0%	120	35.2%
Norway	90	170	41.7%	171	42.0%	179	42.2%
Canada	25	28	10.0%	42	10.1%	19	10.4%
Canada	50	51	20.7%	64	21.1%	34	20.8%
Canada	75	100	31.1%	112	31.7%	82	31.3%
Canada	90	193	36.9%	205	38.0%	188	37.7%
Australia	25	22	11.3%	31	10.3%	13	13.9%
Australia	50	42	22.7%	54	20.6%	28	27.8%
Australia	75	98	34.0%	116	30.8%	70	41.6%
Australia	90	192	40.8%	200	37.0%	167	50.0%

Appendix 2 Table 5a. Key centiles of time-to-chemotherapy at jurisdiction-level, data relate to Appendix 1 Figures 3-6.

			All lung cancers		Stages 1-3 or L-R		Stage 4 or distant	
**Country**	**Jurisdiction**	**Percentile of those treated**	**Days**	**% treated**	**Days**	**% treated**	**Days**	**% treated**
United Kingdom	England	25	22	7.1%	26	7.1%	20	7.9%
United Kingdom	England	50	34	14.2%	41	14.3%	31	15.9%
United Kingdom	England	75	55	21.3%	73	21.4%	47	23.8%
United Kingdom	England	90	96	25.5%	117	25.7%	71	28.6%
United Kingdom	Northern Ireland	25	24	5.7%	31	5.6%	21	6.6%
United Kingdom	Northern Ireland	50	39	11.4%	49	11.3%	34	13.2%
United Kingdom	Northern Ireland	75	66	17.2%	86	16.9%	53	19.8%
United Kingdom	Northern Ireland	90	106	20.6%	126	20.3%	84	23.8%
United Kingdom	Scotland	25	30	6.0%	36	5.5%	28	7.3%
United Kingdom	Scotland	50	49	12.0%	62	10.9%	43	14.6%
United Kingdom	Scotland	75	79	18.1%	100	16.4%	65	21.8%
United Kingdom	Scotland	90	121	21.7%	147	19.7%	98	26.2%
United Kingdom	Wales	25	27	7.2%	31	7.6%	22	7.5%
United Kingdom	Wales	50	42	14.5%	53	15.2%	35	15.1%
United Kingdom	Wales	75	71	21.7%	102	22.8%	52	22.6%
United Kingdom	Wales	90	122	26.0%	146	27.3%	79	27.2%
Norway	Norway	25	13	11.3%	19	10.3%	12	13.4%
Norway	Norway	50	27	22.6%	39	20.7%	22	26.9%
Norway	Norway	75	56	34.0%	76	31.0%	41	40.3%
Norway	Norway	90	97	40.7%	112	37.2%	73	48.4%
Canada	Alberta	25	23	7.5%	32	6.5%	20	8.4%
Canada	Alberta	50	46	14.9%	57	12.9%	38	16.8%
Canada	Alberta	75	85	22.4%	116	19.4%	70	25.2%
Canada	Alberta	90	142	26.8%	164	23.2%	112	30.2%
Canada	British Columbia	25	27	7.1%	33	6.3%	25	8.4%
Canada	British Columbia	50	49	14.2%	58	12.6%	45	16.8%
Canada	British Columbia	75	83	21.3%	97	18.8%	74	25.2%
Canada	British Columbia	90	132	25.6%	146	22.6%	117	30.2%
Canada	Ontario	25	25	10.5%	33	9.4%	21	11.9%
Canada	Ontario	50	48	21.0%	60	18.7%	41	23.8%
Canada	Ontario	75	82	31.4%	101	28.1%	68	35.7%
Canada	Ontario	90	130	37.7%	153	33.7%	106	42.9%
Canada	Saskatchewan-Manitoba	25	39	7.0%	42	6.7%	39	7.5%
Canada	Saskatchewan-Manitoba	50	65	14.0%	68	13.2%	63	15.0%
Canada	Saskatchewan-Manitoba	75	96	21.0%	100	20.0%	92	22.5%
Canada	Saskatchewan-Manitoba	90	163	25.2%	149	24.0%	161	27.0%
Canada	Atlantic Canada	25	26	6.5%	33	6.1%	21	7.3%
Canada	Atlantic Canada	50	49	12.6%	57	12.9%	41	13.4%
Canada	Atlantic Canada	75	85	19.8%	97	19.0%	76	21.4%
Canada	Atlantic Canada	90	143	23.6%	160	22.8%	127	25.2%
Australia	New South Wales	25	21	10.6%	25	9.6%	18	12.2%
Australia	New South Wales	50	39	21.3%	44	19.1%	34	24.3%
Australia	New South Wales	75	64	31.9%	75	28.7%	58	36.5%
Australia	New South Wales	90	107	38.3%	119	34.5%	94	43.8%
Australia	Victoria	25	18	9.9%	*n/a*		*n/a*	
Australia	Victoria	50	36	19.8%				
Australia	Victoria	75	63	29.7%				
Australia	Victoria	90	108	35.6%				

Appendix 2 Table 5b. Key centiles of time-to-radiotherapy at jurisdiction-level, data relate to Appendix 1 Figures 3-6.

			All lung cancers		Stages 1-3 or L-R		Stage 4 or distant	
Country	Jurisdiction	Percentile of those treated	Days	% treated	Days	% treated	Days	% treated
United Kingdom	England	25	30	8.1%	44	9.4%	20	8.0%
United Kingdom	England	50	61	16.2%	80	18.8%	42	16.0%
United Kingdom	England	75	142	24.3%	148	28.2%	133	24.0%
United Kingdom	England	90	200	29.2%	207	33.9%	192	28.8%
United Kingdom	Northern Ireland	25	24	9.4%	36	10.3%	17	9.9%
United Kingdom	Northern Ireland	50	49	18.9%	67	20.6%	35	19.9%
United Kingdom	Northern Ireland	75	117	28.3%	129	30.9%	104	29.8%
United Kingdom	Northern Ireland	90	165	34.0%	179	37.1%	141	35.8%
United Kingdom	Scotland	25	43	8.2%	62	10.0%	27	7.5%
United Kingdom	Scotland	50	78	16.5%	92	19.9%	56	15.1%
United Kingdom	Scotland	75	130	24.7%	137	29.9%	119	22.6%
United Kingdom	Scotland	90	188	29.7%	191	35.9%	182	27.1%
United Kingdom	Wales	25	33	8.6%	49	9.6%	22	8.4%
United Kingdom	Wales	50	72	17.2%	90	19.2%	45	16.8%
United Kingdom	Wales	75	145	25.7%	151	28.9%	132	25.2%
United Kingdom	Wales	90	196	30.9%	200	34.6%	190	30.3%
Norway	Norway	25	28	11.6%	34	11.7%	22	11.7%
Norway	Norway	50	47	23.2%	49	23.3%	44	23.5%
Norway	Norway	75	105	34.8%	94	35.0%	120	35.2%
Norway	Norway	90	170	41.7%	171	42.0%	179	42.2%
Canada	Alberta	25	21	10.8%	38	9.8%	15	11.7%
Canada	Alberta	50	40	21.6%	60	19.7%	27	23.4%
Canada	Alberta	75	89	32.4%	112	29.5%	59	35.2%
Canada	Alberta	90	170	38.8%	187	35.4%	158	42.2%
Canada	British Columbia	25	21	10.7%	41	9.9%	14	12.2%
Canada	British Columbia	50	43	21.3%	62	19.9%	30	24.4%
Canada	British Columbia	75	83	32.0%	102	29.8%	60	36.7%
Canada	British Columbia	90	151	38.4%	165	35.8%	138	44.0%
Canada	Ontario	25	33	9.9%	42	11.5%	23	8.8%
Canada	Ontario	50	56	19.7%	64	22.9%	42	17.7%
Canada	Ontario	75	100	29.6%	102	34.4%	94	26.5%
Canada	Ontario	90	179	35.5%	168	41.3%	188	31.8%
Canada	Saskatchewan-Manitoba	25	25	10.2%	48	8.4%	16	11.8%
Canada	Saskatchewan-Manitoba	50	49	20.0%	78	16.8%	33	23.1%
Canada	Saskatchewan-Manitoba	75	101	29.0%	151	25.6%	62	33.5%
Canada	Saskatchewan-Manitoba	90	204	35.2%	222	30.2%	174	41.1%
Canada	Atlantic Canada	25	21	10.8%	35	10.6%	13	11.1%
Canada	Atlantic Canada	50	42	21.8%	55	21.0%	25	22.0%
Canada	Atlantic Canada	75	91	32.1%	98	31.1%	63	33.4%
Canada	Atlantic Canada	90	178	38.6%	174	37.7%	167	40.3%
Australia	New South Wales	25	20	12.1%	31	10.3%	13	13.9%
Australia	New South Wales	50	41	24.1%	54	20.6%	28	27.8%
Australia	New South Wales	75	94	36.2%	116	30.8%	70	41.6%
Australia	New South Wales	90	185	43.4%	200	37.0%	167	50.0%
Australia	Victoria	25	22	10.2%	*n/a*		*n/a*	
Australia	Victoria	50	42	20.4%				
Australia	Victoria	75	98	30.6%				
Australia	Victoria	90	192	36.7%				
