# Supplementary material for: Chemotherapy and radiotherapy use in patients with lung cancer in Australia, Canada, the UK and Norway 2012–2017: an ICBP population-based study
Source: BMJ Oncol. 2025 Jul 11;4(1):e000800. doi: 10.1136/bmjonc-2025-000800 (PMC12258358; doi:10.1136/bmjonc-2025-000800)
Supplement: online supplemental file 3 [file bmjonc-4-1-s003.pdf]

APPENDIX 3. ADDITIONAL DISCUSSION AND CONTEXT.

Contents

Section 1. Consideration of missing stage in our data.. 1

Section 2. Management of lung cancer during the study period (‘mid-2010s’)....................... 2

Bibliography for Appendix 3 3

Section 1. Consideration of missing stage in our data

Except for Victoria, the proportion of patients with missing stage was relatively small (14% or lower). Missing stage data may introduce bias into stage-specific treatment use estimates, because if patients with more advanced cancer who were too ill to receive treatment were more likely to have missing stage in some jurisdictions, this would increase apparent treatment use in those with known advanced stage cancer (through a strong *missing not at random* mechaniam). One sign this might be a concern would be if the proportion of patients with no recorded stage who received treatment remained close to zero despite differences in the proportion of patients with recorded stage. We do not see this. In jurisdictions with more missing stage information (e.g., New South Wales, Norway), the proportion of patients with no recorded stage who receive treatment is reassuringly similar to the overall proportion.

While this reassures that treatment use in patients with known stage should be comparable between jurisdictions, we should otherwise pay little attention to recorded treatment use in patients with no recorded stage. In essence, the no recorded stage group is a mix of:

(a) patients with truly unstageable cancer;
(b) patients whose cancer could be staged but they were too ill for staging investigation; and
(c) patients whose cancer was staged but the information was not recorded by the registry.

In jurisdictions with low proportions of missing stage data, the missing stage group is dominated by patients of type (a) and (b), and the proportion receiving treatment is typically low (e.g., Alberta, where the proportion of the 119 patients with missing stage information who received chemotherapy was 14%, vs 43% for all 9,408 patients). In jurisdictions with higher proportions of missing stage, there are more patients from group (c) - for example, in Norway, 44% of the 1,414 patients with no recorded stage received treatment, vs 46% for all 11,547 patients).

Section 2. Management of lung cancer during the study period ('mid-2010s')

During the study period, for non-small cell lung cancer, clinical management would have generally entailed:

a) For non-advanced/resectable disease, surgical excision with curative intent, possibly combined with adjuvant chemotherapy (depending on disease spread) and adjuvant radiotherapy (e.g., for positive resection margins) [1]. Conventionally fractionated radiotherapy was an established alternative to lung excision surgery during the study period, particularly for patients with high comorbidity burden; additionally, stereotactic radiotherapy was being introduced into clinical practice during the 2010s, with variable adoption speed;

b) For advanced/non-resectable non-small lung cancer, palliative use of chemotherapy, with radiotherapy for symptomatic disease [2].

c) For small cell lung cancer, clinical management would have generally entailed use of chemotherapy with radiotherapy for limited stage disease, and chemotherapy without radiotherapy for extensive stage [3].

d) A proportion of patients with lung cancer of either type whose disease was sufficiently advanced and who had poor performance status would have been managed without use of cancer-directed treatments.

Beyond disease stage and patient factors, currently, the management of lung cancer depends on tumour characteristics such as histological type and tumour marker profiling to guide the use of targeted drug treatments or immunotherapy, which have expanded substantially since the mid-2010s. The management of more localised disease has also evolved, for example with increasing use of stereotactic radiotherapy.

Bibliography for Appendix 3

1 Vansteenkiste J, De Ruysscher D, Eberhardt WEE, Lim E, Senan S, Felip E, Peters S. Early and locally advanced non-small-cell lung cancer (NSCLC): ESMO Clinical Practice Guidelines for diagnosis, treatment and follow-up†. *Annals of Oncology*. 2013;24:vi89–98. doi: 10.1093/annonc/mdt241

2 Reck M, Popat S, Reinmuth N, De Ruysscher D, Kerr KM, Peters S. Metastatic non-small-cell lung cancer (NSCLC): ESMO Clinical Practice Guidelines for diagnosis, treatment and follow-up†. *Annals of Oncology*. 2014;25:iii27–39. doi: 10.1093/annonc/mdu199

3 Früh M, De Ruysscher D, Popat S, Crinò L, Peters S, Felip E. Small-cell lung cancer (SCLC): ESMO Clinical Practice Guidelines for diagnosis, treatment and follow-up†. *Annals of Oncology*. 2013;24:vi99–105. doi: 10.1093/annonc/mdt178
